# MicroRNAs Are Part of the Regulatory Network that Controls EGF Induced Apoptosis, Including Elements of the JAK/STAT Pathway, in A431 Cells

**DOI:** 10.1371/journal.pone.0120337

**Published:** 2015-03-17

**Authors:** Ibrahim Alanazi, Peter Hoffmann, David L. Adelson

**Affiliations:** 1 School of Biological Sciences, The University of Adelaide, Adelaide, South Australia, Australia; 2 Zhendong Australia—China Centre for Molecular Chinese Medicine, The University of Adelaide, Adelaide, South Australia, Australia; National Institutes of Health, UNITED STATES

## Abstract

MiRNAs are known to regulate gene expression and in the context of cancer have been shown to regulate metastasis, cell proliferation and cell death. In this report we describe potential miRNA regulatory roles with respect to induction of cell death by pharmacologic dose of Epidermal Growth Factor (EGF). Our previous work suggested that multiple pathways are involved in the induction of apoptosis, including interferon induced genes, cytokines, cytoskeleton and cell adhesion and TP53 regulated genes. Using miRNA time course expression profiling of EGF treated A431 cells and coupling this to our previous gene expression and proteomic data, we have been able to implicate a number of additional miRNAs in the regulation of apoptosis. Specifically we have linked miR-134, miR-145, miR-146b-5p, miR-432 and miR-494 to the regulation of both apoptotic and anti-apoptotic genes expressed as a function of EGF treatment. Whilst additional miRNAs were differentially expressed, these had the largest number of apoptotic and anti-apoptotic targets. We found 5 miRNAs previously implicated in the regulation of apoptosis and our results indicate that an additional 20 miRNAs are likely to be involved based on their correlated expression with targets. Certain targets were linked to multiple miRNAs, including PEG10, BTG1, ID1, IL32 and NCF2. Some miRNAs that target the interferon pathway were found to be down regulated, consistent with a novel layer of regulation of interferon pathway components downstream of JAK/STAT. We have significantly expanded the repertoire of miRNAs that may regulate apoptosis in cancer cells as a result of this work.

## Introduction

The epidermal growth factor receptor (HER1) and HER2 are members of the receptor tyrosine kinase type one (RTK-I) family. Activation of these receptors plays crucial roles in survival, migration [1], development, proliferation and differentiation [2]. Epidermal growth factor (EGF) and other ligands binds to HER1 and forms homodimers or hetrodimers with HER2 [2]. Activation of HER 1 signalling usually induces either proliferation or cell survival [3], a fact that has resulted in a strategy that targets RTK-I mediated survival signals in cancer [4–10]. However, there are significant and reproducible reports of EGF inducing apoptosis in cell lines such as A431 that over-express RTK-I [11–17]. The gene expression and protein profiles of these cells were characterised after EGF treatment in order to globally survey the induction of apoptosis[18]. As a result of this study, multiple novel regulatory sub-networks /pathways mediating EGF induced apoptosis were observed. Because MicroRNAs (miRNAs) are known to regulate pathways governing cancer cell survival, differentiation and metastasis we expected that they might be important regulators of the above gene expression/protein sub-networks.

MiRNAs are a major class of gene regulators that participate in large scale modulation of gene expression [19,20]. MiRNAs are noncoding RNA molecules transcribed as nascent primary miRNA transcripts (Pri-mRNA) by RNA polymerase II. After Pri-mRNA transcripts are processed into miRNA precursors (pre-miRNA) by Drosha and Pasha, pre-mRNAs are exported from the nucleus to the cytoplasm by exportin 5 and subsequently processed by Dicer into mature miRNAs. These miRNAs can incorporate into the RNA-induced silencing complex (RISC) to target protein-coding messenger RNA (mRNA) and inactivate gene expression by either mRNA degradation or inhibition of translation [21–24]. MicroRNAs have recently been described as novel tools for cancer diagnosis and may help identify and validate cancer targets [22–24]. A recent report shows that some miRNAs such as miR-17–5p/miR-20a can suppress breast cancer cell proliferation via a conserved 3'-UTR miRNA-binding site [21]. In addition, miRNAs regulate the expression of target genes that can act as a controllers of growth, development, differentiation, cancer development and progression [25–27]. Furthermore, miRNAs are key regulators of apoptosis in cancer because they can act as positive regulators of apoptosis by acting as negative regulators of anti-apoptotic mRNAs and as negative regulators of apoptosis by acting as negative regulators of pro-apoptotic mRNAs [28].

Recently, we found that when a high dose of EGF is used to induce apoptosis in A431 cells that multiple gene expression and protein expression sub-networks are stimulated, including interferon response, cytokine signalling, cytoskeleton and cell adhesion pathways. Specifically we found that between 3h and 12h post-EGF treatment significant changes in gene expression occurred in the lead up to caspase induced apoptosis. In order to provide a deeper understanding of these regulatory mechanisms, it is essential to build an integrated genetic regulatory network that includes post-transcriptional (miRNA) regulatory interactions.

In this study, we have identified novel interaction regulatory networks based on the crosstalk between miRNAs and mRNA/protein that resulted in the induction of apoptosis in A431 cells. We have also identified a number of miRNAs that may play an important role in the regulation of apoptosis in A431 cells after EGF treatment.

## Materials and Methods

### Cell culture and treatment

A431 (The epidermoid carcinoma cell line) cell line was purchased from the ATTC (Manassas,VA). Cells were cultured at 37°C in a humidified 5% CO2 atmosphere. Dulbecco’s modified Eagle’s medium, 10% fetal calf serum, and 4 mM L-glutamine (Cambrex Bio Science). After 24h serum-free, in addition to control (no EGF treatment), cells were treated with 100 ng EGF concentrations and sampled at 3h and 12h after treatment [18].

### Microarray analysis for miRNA expression in A431 cell line after EGF treatment

Total RNA were extracted from A431 cells using the mirVana miRNA isolation kit (Ambion, Inc., Austin, TX, USA), according to the manufacturer’s instruction. RNA’s concentration was determined by Nanodrop (ND1000) spectrophotometer (NanoDrop Technologies, Inc., Wilmington, DE) and its purity was determined by bioanalyzer 2100 (Agilent Technologies, Santa Clara, CA). We performed comprehensive expression profiling of mature miRNA using the Affymetrix GeneChip 2.0 miRNA Array (Affymetrix, Santa Clara, CA). Labeling of 1μg of total RNA samples was performed using the FlashTag Biotin RNA labeling kit (Genisphere Inc., Hatfield, PA) according to manufacturer’s instruction. Using an Affymetrix GeneChip Fluidics Station 450, the arrays were washed and stained and then scanned with an Affymetrix GeneChip scanner (3000 7G). The CEL files containing the raw Affymetrix 2.0 microRNA array intensity data were processed using the Bioconductor tools that were used for image analysis, data import, background adjustment, normalization (based on RMA algorithm), summarization, and quality assessment [29,30]. Cancer cell line (no EGF application) and two time points after EGF application (3h and 12 h) were assumed as treatments. For each time points, RNAs were extracted from 3 samples as replications. Differential gene expression analyses were performed using linear regression models in the limma package [31]. For transcript analysis (microarray data), Log2 ratio values of each time point verses control (cancer cell line) were assumed as fold change. Those having a Bayesian t-test with adjusted p-value with false discovery rate <0.1 and fold change ≥ +1.5 or fold change ≤ -1.5 were accepted as significant differentially expressed genes.

### Microarray analysis for mRNA expression in A431 cells after EGF treatment

Cell culture, mRNA isolation and microarray analysis described in [18].

### Proteomic analysis of A431 cells after EGF treatment

Cell culture, protein isolation and proteomic profiling described in [18].

### Target prediction of significantly expressed microRNA

Several methods were used to predict targets of significantly expressed microRNAs including miRBase (http://www.mirbase.org/), and TargetScan (www.targetscan.org/). Targets of significantly expressed microRNAs were then matched with significantly expressed genes (using our microarray data) and significantly expressed proteins (using our proteomics data) in order to obtained only genes and proteins in our data potentially regulated by microRNA(s).

### Reactome functional interactions (FI) Cytoscape

Correlations between genes/proteins involved in the same functional interactions (FIs) were carried out using The Reactome FI Cytoscape plugin (Pearson correlation). The correlations were then used as input for the Reactome FI Cytoscape plugin (Markov cluster algorithm) to generate a sub-network for a list of selected network modules based on module size and average correlation [32].

### Gene and protein classification according to gene ontology

PANTHER (Protein ANalysis THrough Evolutionary Relationships) classification software was used to assign protein classes to identified genes and proteins [33].

## Results

### MiRNA expression

**Fig 1:**
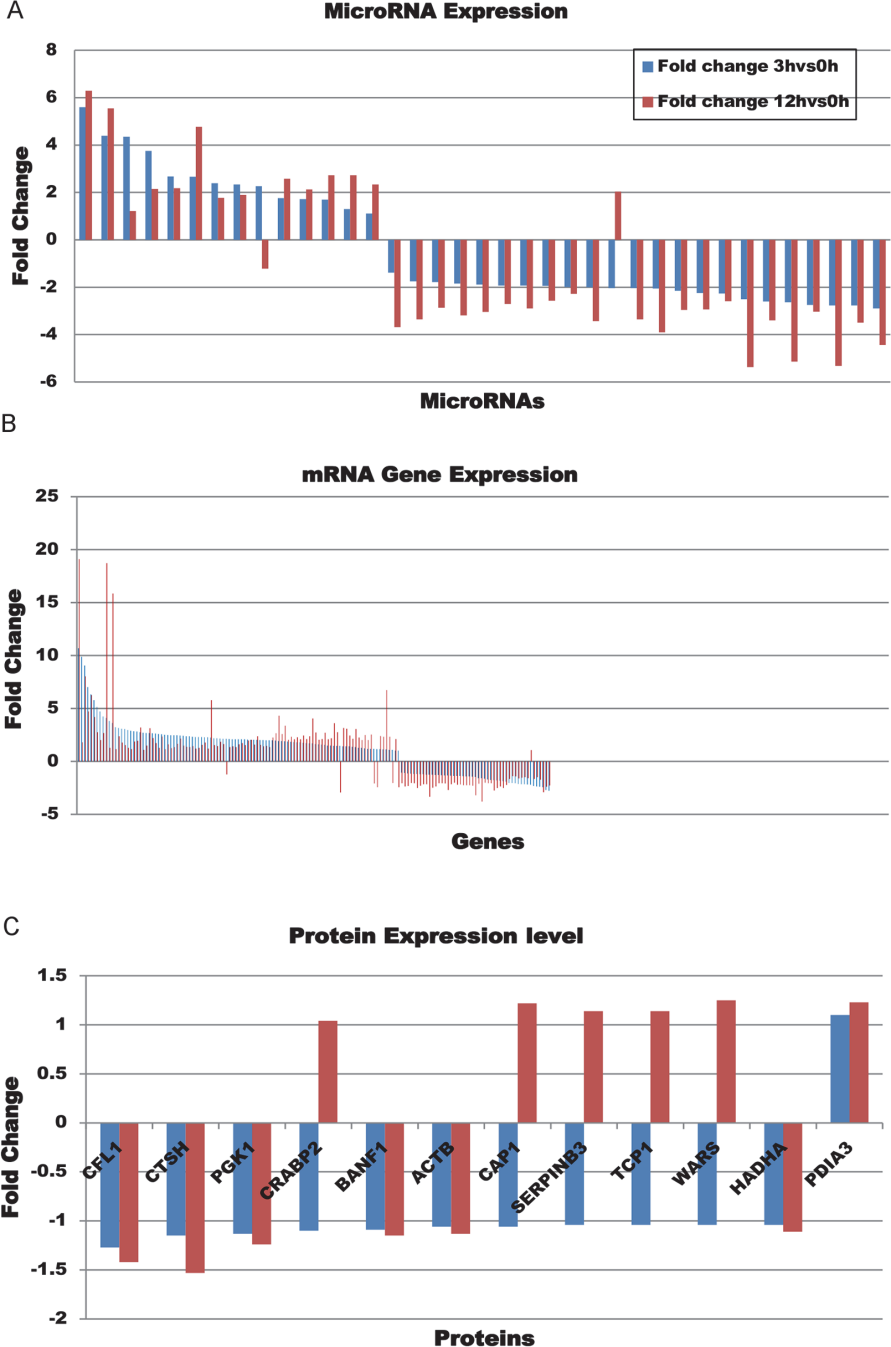
MicroRNAs, mRNA and protein expression at 3h vs 0h and 12h vs 0h fold change. Blue bars indicate 3h vs 0h fold change and red bars indicate 12h vs 0h fold change. (A) miRNA expression levels expressed as fold changes with respect to pre-treatment levels. (B) mRNA gene expression expressed as fold change with respect to pre-treatment levels. (C) Protein expression expressed as fold change with respect to pre-treatment levels.

Our analysis of the miRNA time course expression values, based on a False Discovery Rate of 10% yielded 37 miRNAs that were differentially expressed between the time of EGF treatment and either 3 hours or 12 hours post-EGF treatment (Table 1). Of the 37 differentially expressed miRNAs about two thirds decreased at 3 or 12 hours post EGF treatment, and the remaining third increased in expression. The changes between 0h and 3h and 0h and 12h were very consistent with only two miRNAs flipping from either increased expression at 3h to decreased expression at 12h or vice versa (Fig. 1A). The two miRNAs that flipped expression pattern with respect to untreated cells were miR-432 (-2.04 fold change at 3h, 2.03 fold change at 12h) and miR-499–5p (2.26 at 3h, ‒1.22 at 12h).

**Table 1:** 37 MicroRNAs with significant changes to expression.

MicroRNA	Apoptotic		Anti-apoptotic		Fold change 3h vs 0h	Fold change 12h vs 0h	P-Value	mRNA (M) / Protein (P) Target in networks
	3h vs 0h	12h vs 0h	3h vs 0h	12h vs 0h				
hsa-miR-1228-star_st	0	0	0	0	‒2.06	‒3.9	0.02	‒
hsa-miR-146b-3p_st	3(1)*	5	1(1)*	1	5.6	6.29	0.02	M
hsa-miR-1908_st	0	0	0	0	‒2	‒3.43	0.02	-
hsa-miR-1909_st	1	1	1	0	‒2.77	‒5.32	0.02	M-P
hsa-miR-3180–3p_st	0	0	0	0	‒2.51	‒5.37	0.02	-
hsa-miR-4321_st	0	0	0	0	‒1.75	‒3.36	0.02	-
hsa-miR-596_st	2	2	0	1	‒2.6	‒3.4	0.02	P
hsa-miR-92a-1-star_st	0	0	1	0	4.39	5.55	0.02	-
hsa-miR-145_st	3	1	1	4	2.67	2.18	0.03	M-P
hsa-miR-29b-1-star_st	0	1	3	0	3.75	2.15	0.03	M-P
hsa-miR-432_st	1	2	1	2	‒2.04	2.03	0.03	M-P
hsa-miR-663_st	2	5	0	1	‒1.85	‒3.19	0.03	M
hsa-miR-149-star_st	1	1	0	0	‒1.93	‒2.71	0.04	P
hsa-miR-602_st	0	1(1)*	1	2(1)*	‒2.77	‒3.49	0.04	M
hsa-miR-762_st	1	0	1	0	‒2.04	‒3.36	0.04	M
hsa-miR-134_st	2	4(1)*	1	4(1)*	2.39	1.77	0.05	M-P
hsa-miR-222-star_st	0	0	0	0	4.35	1.22	0.05	M
hsa-miR-665_st	0	0	3	2	‒2.75	‒3.03	0.05	M
hsa-miR-1231_st	2	0	0	0	‒2.63	‒5.14	0.06	-
hsa-miR-1469_st	0	0	0	0	‒1.89	‒3.04	0.06	-
hsa-miR-3185_st	2	2	0	0	‒2.9	‒4.44	0.07	M
hsa-miR-3195_st	0	0	0	0	‒1.99	‒2.28	0.07	-
hsa-miR-92b-star_st	0	0	0	0	‒2.24	‒2.94	0.07	-
hsa-miR-1972_st	2(1)*	2	1(1)*	1	1.3	2.73	0.08	M
hsa-miR-21-star_st	2	0	1	0	2.34	1.9	0.08	-
hsa-miR-23a-star_st	0	0	0	0	1.76	2.58	0.08	-
hsa-miR-27a-star_st	0	0	0	0	1.72	2.13	0.08	
hsa-miR-2861_st	1	1	0	0	‒2.15	‒2.96	0.08	M
hsa-miR-3178_st	0	0	0	0	‒1.78	‒2.86	0.08	-
hsa-miR-3179_st	0	0	0	0	1.11	2.34	0.08	-
hsa-miR-3181_st	0	0	0	0	‒1.93	‒2.9	0.08	-
hsa-miR-4304_st	0	0	0	0	2.66	4.77	0.08	-
hsa-miR-494_st	9	4(1)*	3	4(1)*	‒1.38	‒3.68	0.08	-
hsa-miR-499–5p_st	4	4	3	4	2.26	‒1.22	0.08	M
hsa-miR-638_st	1	0	1	1	‒1.94	‒2.57	0.09	-
hsa-miR-146b-5p_st	2(1)*	1(1)*	3(1)*	5(1)*	1.7	2.73	0.1	M
hsa-miR-675_st	0	3	0	1	‒2.27	‒2.59	0.1	M

* () brackets indicate targets that have been annotated as both apoptotic and anti-apoptotic.

### MiRNA targets

Because miRNAs can target multiple mRNAs we expected the total number of target genes regulated by these miRNAs to be greater in number. In order to identify likely target transcripts we identified all possible target genes using both TargetScan and miRBase and then determined which targets were differentially expressed within our gene-expression and proteomic data. As a result of this analysis we identified 165 unique differentially expressed genes at both 3 and 12h post EGF treatment in both our gene-expression data and proteomic data. In order to identify which targets might regulate apoptosis, we carried out an exhaustive manual literature search of all differentially expressed targets in order to annotate them with respect to possible pro or anti-apoptotic functions. Within the gene expression data set, 77 targets were differentially expressed at 3h post EGF treatment, with 17 of these annotated as having anti-apoptotic function, 20 annotated as pro-apoptotic and 2 annotated as both pro and anti-apoptotic. At 12h post EGF treatment there were 99 differentially expressed targets (some of which were shared with 3h post EGF) and 17 of these were anti-apoptotic, 19 pro-apoptotic and 1 both anti and pro-apoptotic (S1 Table). Within the proteomic data set we identified the same 12 targets at both 3h and 12 post EGF and of these 1 was anti-apoptotic and 6 were pro-apoptotic (S2 Table).

Because most of the differentially expressed targets had no previous link to apoptosis we used PANTHER [33] to classify our targets from both gene-expression and proteomic data with respect to function (Table 2). Our gene-expression targets fell into 25 classes including signalling molecules, receptors, transmembrane receptors, transcription factors, cytoskeleton, cell-adhesion and extra cellular matrix. Our protein targets fell into 12 classes, including cytoskeleton and chaperone, a class not found in the gene-expression data. In order to visualise which targets might be acting together to regulate apoptosis we constructed correlation networks for gene-expression and proteomic data [18] and then added miRNAs based on their known targets in the networks.

**Table 2:** Protein functional classes for gene expression and protein levels.

	Gene expression Protein classes		Protein levels protein classes	
protein-class	3h vs 0h	12h vs 0h	3h vs 0h	12h vs 0h
calcium-binding protein	+	+	‒	‒
cell adhesion molecule	+	+	‒	‒
cell junction protein	+	+	‒	‒
cytoskeletal protein	+	+	+	+
enzyme modulator	+	+	+	+
extracellular matrix protein	+	+	‒	‒
hydrolase	+	+	+	+
isomerase	+	-	+	+
kinase	+	+	+	+
ligase	+	+	+	+
lyase	+	-	+	+
nucleic acid binding	+	+	‒	‒
oxidoreductase	+	+	+	+
phosphatase	+	+	‒	‒
protease	+	+	+	+
receptor	+	+	‒	‒
signaling molecule	+	+	‒	‒
structural protein	+	+	‒	‒
transcription factor	+	+	‒	‒
transfer/carrier protein	+	-	+	+
transferase	+	+	+	+
transmembrane receptor regulatory/adaptor protein	+	-	‒	‒
transporter	+	+	‒	‒
defense/immunity protein	-	+	‒	‒
membrane traffic protein	-	+	‒	‒
chaperone	-	-	+	+

### MiRNAs and network regulation

**Fig 2:**
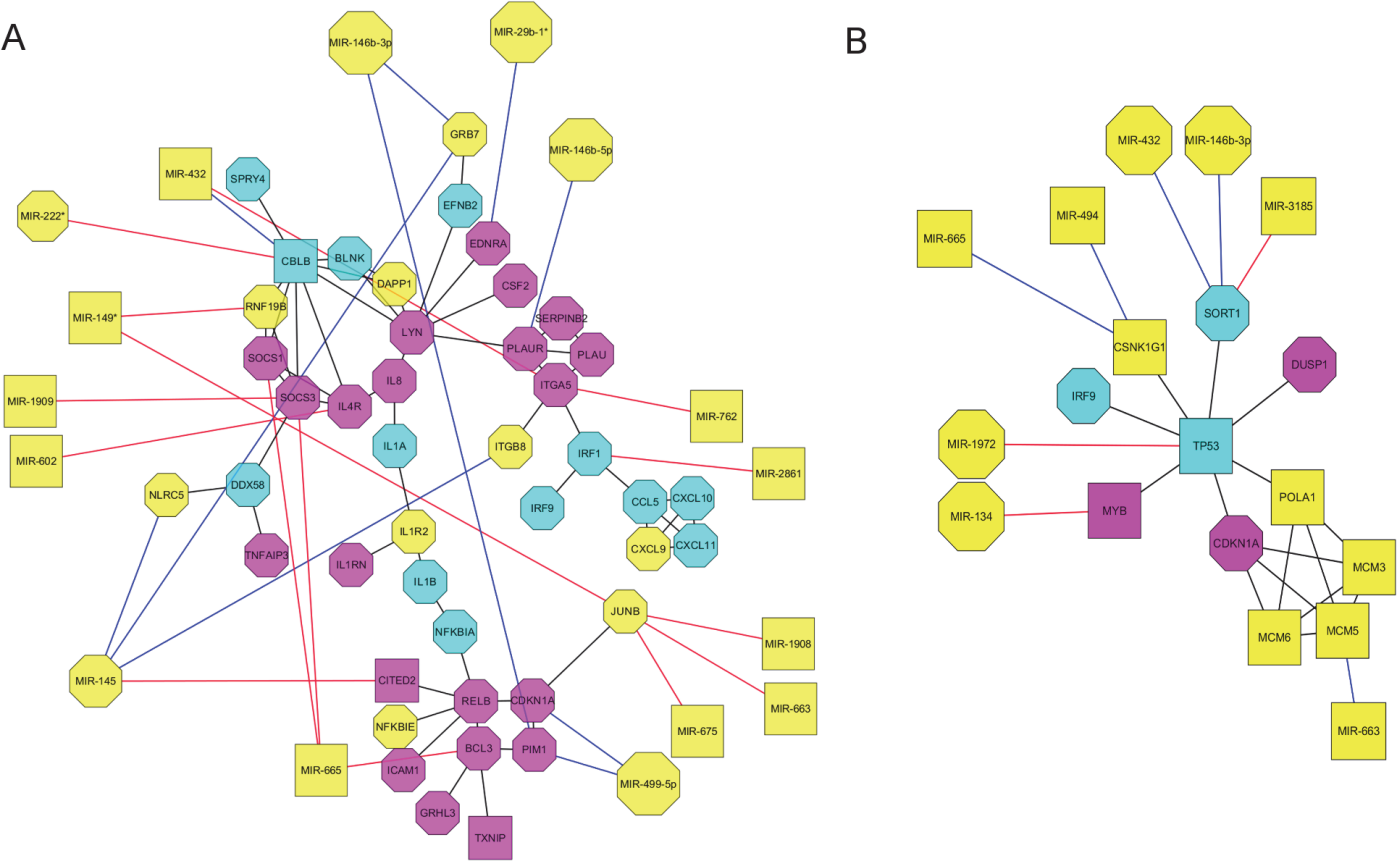
Correlation gene expression networks based on single time point gene expression data. (A) pairwise gene expression data 0h-3h. (B) pairwise gene expression data 0h-12h. Anti-apoptotic genes coloured in pink, pro-apoptotic genes coloured in light blue, genes with no reported involvement in apoptosis coloured in yellow. Octagonal shape indicates up-regulated genes. Squares for down-regulated genes. MicroRNAs negatively correlated with respect to target have red edges while MicroRNAs positively correlated with respect to target have blue edges.

We used the Reactome Functional Interaction plug-in from Cytoscape [32] to construct correlation networks based on gene-expression or proteomic data. The correlation networks for gene expression differed significantly in complexity between 3h and 12h post EGF treatment, with a reduction in network complexity as judged by fewer nodes. As reported previously [18], at 3h (Fig. 2A) we observed correlated changes in gene expression for a variety of mRNAs, including immediate early response genes, cytokines, cytokine signalling suppressors, interferon response genes and cell adhesion genes. When we added our miRNAs to the network based on the presence of known targets, we found 15 miRNA targets regulated by 16 miRNAs, 6 of which regulated 2 or 3 targets for a total of 24 edges connecting miRNAs to targets. In addition, 6 targets were regulated by more than one miRNA and miR-145 had 4 targets. Furthermore, we found that a greater proportion of anti-apoptotic genes were targets for miRNAs. Specifically, 9 of 21 anti-apoptotic genes were miRNA targets compared to 2 of 13 pro-apoptotic genes. Of the 6 targets regulated by more than one miRNA, 5 were anti-apoptotic (SOCS3 (suppressor of cytokine signaling 3), PM1(transmembrane protein 11), JUNB (jun B proto-oncogene),ITGA5 (integrin, alpha 5) and GRB7 (growth factor receptor-bound protein 7)) with JunB an anti-apoptotic target for 4 miRNAs. CBLB (Cbl proto-oncogene, E3 ubiquitin protein ligase B) was the only pro-apoptotic gene linked to 2 miRNAs.

Because negatively correlated miRNA/target pairs provide more compelling evidence of potential regulation we looked for miRNA/mRNA pairs that had negatively correlated fold changes. We found 14 miRNA/target edges that were consistent with 14 negatively correlated miRNAs and 8 mRNAs, of which 2 were pro-apoptotic and 6 were anti-apoptotic. However, at 12 h post-EGF treatment (Fig. 2B) the network was much simpler with only 8 miRNAs connected by 8 edges to 5 targets (of 11 genes). Some of the edges were more consistent with a regulatory function, and 3 described miRNA/mRNA relationships that were negatively correlated. Two of these 3 genes were pro-apoptotic targets, SORT1 (sortilin 1) and TP53 (tumor protein p53), with SORT1 linked to 3 miRNAs. In fact, SORT1 mRNA actually increased from 3hrs in spite of being targeted by 3 miRNAs, 2 of which increased and one decreased from 3 hrs. The remaining mRNA was an anti-apoptotic target, MYB (v-*myb* myeloblastosis viral oncogene homolog (avian), targeted by a single miRNA (miR-134). It is worth pointing out that miR-663 targeted MCM5 (minichromosome maintenance complex component 5), which encodes a chromatin binding protein that may regulate the cell cycle [34]. MCM5 is part of a smaller, highly connected cluster that includes POLA1 (polymerase (DNA directed), alpha 1, catalytic subunit), MCM3 (minichromosome maintenance complex component 3) and MCM6 (minichromosome maintenance complex component 6) all involved in DNA replication.

**Fig 3:**
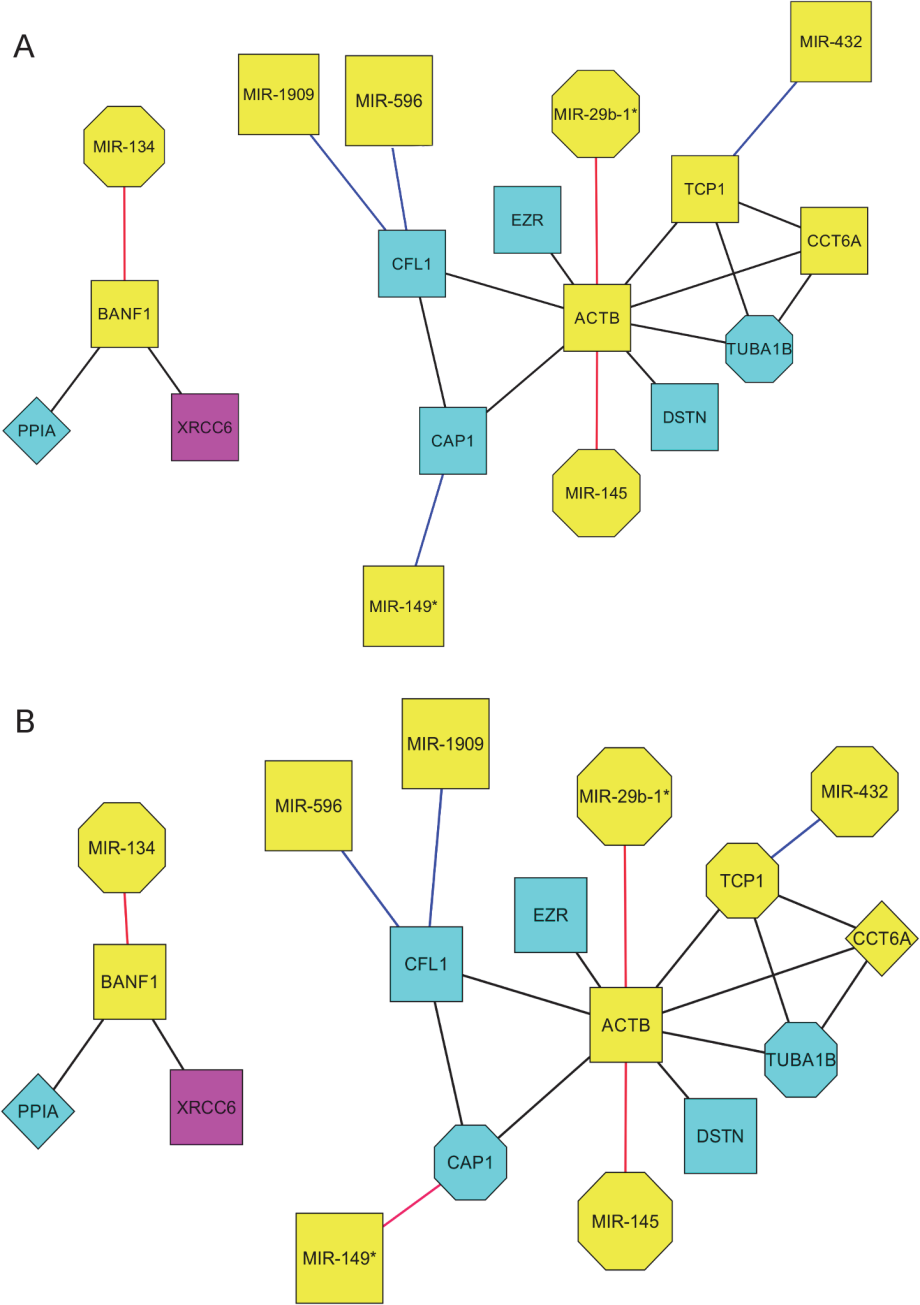
Expression correlation network based on Proteomics data. There was no difference in the networks identified using pairwise proteomics data based on single time points from 3 or 12h after EGF treatment. Anti-apoptotic genes coloured in pink, pro-apoptotic genes coloured in light blue, genes with no reported involvement in apoptosis coloured in yellow. Octagonal shape indicates up-regulated genes. Squares for down-regulated genes. MicroRNAs negatively correlated with respect to target have red edges while MicroRNAs positively correlated with respect to target have blue edges. Diamonds indicate targets with isoforms that are both up and down regulated. Represented protein classes include: chaperone (TCP1 and CCT6A), cytoskeletal protein (EZR, DSTN, CAP1, TUBA1B and ACTB), isomerase (PPIA) and nucleic acid binding (XRCC6).

We also found miRNAs targets in our protein expression correlation network (Fig. 3). Unlike the gene expression sub-networks, at 3h (Fig. 2A) and 12h (Fig. 2B) post EGF treatment the same sub-network topologies were found for protein expression. Overall we observed 7 miRNAs linked by 7 edges to 5 targets. At 3 h post-EGF all targets decreased in abundance, while at 12 h, 2 targets had increased in abundance. One sub-network, which included MIR-134 as a regulator of BANF1 (Barrier to autointegration factor 1), thought to function as a regulator of nuclear assembly, was correlated with the expression of PPIA (peptidylprolyl isomerase A (cyclophilin A)) an enzyme known to catalyse protein folding and known to participate in the induction of apoptosis [35], and XRCC6 (x-ray repair cross complementing protein 6) a DNA binding helicase capable of interacting with transcription factors but also known to protect cells against oxidative stress and apoptosis [36]. The other sub-network included primarily cytoskeletal proteins, with 4 targets, three of which, CAP1 (CAP, adenylate cyclase-associated protein 1), CFL1 (Cofilin 1) and ACTB (Actin, beta) are cytoskeletal components, and the remaining target TCP1 is known to regulate the folding of ACTB and TUBA1B (Tubulin, alpha 1b). Both ACTB and CFL1 were targets for two miRNAs, and CAP1, CFL1 and ACTB are known to be part of an interaction network regulating cytoskeletal polymerisation. Five of the 8 proteins in this sub-network are known to be pro-apoptotic and of these 8, both CAP1 and TCP1 increased in abundance from 3h to 12 h post EGF.

### Overlap of gene-expression and proteome miRNA targets

The overlap between the gene-expression data and the proteome data is small, with only 4 loci common to both sets, accounting for about 8% of the proteins and about 1% of the mRNAs ([18]. However, the proportion of miRNAs that had targets in both the gene-expression data and the proteome data was higher. Of the 21 miRNAs (Table 3) with targets in either the gene-expression or proteome sub-networks, 14 had targets found only in gene-expression sub-networks, 1 had a target only found in protein sub-networks and 6 had targets that were found in both gene-expression and protein sub-networks. However, no miRNA that had targets in both sets shared common targets across the two sets.

**Table 3:** Summary of miRNAs and their relationship to networks from gene expression and proteomic data.

microRNA	Gene expression		Protein level	
^1^ ID	3hvs0h fold change^2^	12hvs0h fold change^2^	3hvs0h fold change^3^	12hvs0h fold change^3^
hsa-miR-134_st	−	+	+	+
hsa-miR-145_st	+	−	+	+
hsa-miR-146b-3p_st	+	+	−	−
hsa-miR-146b-5p_st	+	−	−	−
hsa-miR-149-star_st	+	−	+	+
hsa-miR-1908_st	+	−	−	−
hsa-miR-1909_st	+	−	+	+
hsa-miR-1972_st	−	+	−	−
hsa-miR-222-star_st	+	−	−	−
hsa-miR-2861_st	+	−	−	−
hsa-miR-29b-1-star_st	+	−	+	+
hsa-miR-3185_st	−	+	−	−
hsa-miR-432_st	+	+	+	+
hsa-miR-494_st	−	+	−	−
hsa-miR-499–5p_st	+	−	−	−
hsa-miR-596_st	−	−	+	+
hsa-miR-602_st	+	−	−	−
hsa-miR-663_st	+	+	−	−
hsa-miR-665_st	+	+	−	−
hsa-miR-675_st	+	−	−	−
hsa-miR-762_st	+	−	−	−

^1^ Significant fold change with adjusted p value with FDR 0.1 as can be seen in S1 Table.

^2^ Significant fold change with p value < 0.05 as can be seen in S2 Table.

^3^ Significant fold change based on *DeCyder software* one-way *ANOVA analysis* of p value < 0.05.

### Negatively correlated miRNA/mRNA pairs

If miRNAs were regulating either mRNA or protein expression in our experiment, we might expect that miRNAs that increased in expression would be associated with targets that decreased in expression and *vice versa*, leading to negatively correlated miRNA/target pairs. In our data a number of miRNAs showed negatively correlated changes in expression with targets known to regulate apoptosis (Tables 4 and 5), so these miRNAs and targets appear to be better candidates for further investigation. Within this negatively correlated miRNA/target set BTG1 (B-Cell Translocation Gene 1, Anti-Proliferative), which has anti-proliferative function and has been shown to induce apoptosis [37], increased in expression while the two potentially regulatory miRNAs (miR-596 and miR-1231) decreased in expression. ID1 (inhibitor of DNA binding 1, dominant negative helix-loop-helix protein) has been reported to induce apoptosis via P38 MAP Kinase [38] and increased in expression while the three miRNAs that target it (miR-494, miR-602 and miR-663) decreased in expression. IL32 (interleukin 32) also increased in expression and is known to induce apoptosis and was potentially regulated by miR-663 and miR-675, which decreased in expression. Finally, NCF2 (neutrophil cytosolic factor 2) also increased in expression and is known to induce apoptosis and was potentially regulated by miR-494 and miR-2861. Interestingly, IRF1 (interferon regulatory factor 1) a transcription factor of the interferon induced pathway associated with apoptosis also increased in expression and was targeted by Mir-2861 which decreased in expression. One particular target with anti-apoptotic activity, PEG10 (paternally expressed 10), decreased in expression while the three miRNAs (miR-145, miR-432 and—miR-1972) capable of targeting it increased in expression.

**Table 4:** Differentially regulated interaction between miRNAs and genes where fold changes are negatively correlated, in gene expression data.

**A. MicroRNA gene expression with their apoptotic or anti-apoptotic target mRNA gene expression at 3h, sorted by target**.				
miRNA ID	miRNA fold change 3h vs 0h	Target gene	mRNA fold Change 3h vs 0h	Apoptotic or anti apoptotic Function
hsa-miR-3185	‒2.9	ARHGEF2	2.42	Apoptotic
hsa-miR-665	‒2.75	BCL3	3.81	Anti-apoptotic
hsa-miR-499-5p	2.26	BHLHE41	‒2.37	Anti-apoptotic
hsa-miR-596	‒2.6	BTG1	2.09	Apoptotic
hsa-miR-1231	‒2.63	BTG1	2.09	Apoptotic
hsa-miR-145	2.67	CITED2	‒2.21	Anti-apoptotic
hsa-miR-3185	‒2.9	DCUN1D3	3.08	Apoptotic
hsa-miR-145	2.67	FBXO32	‒2.77	Apoptotic
hsa-miR-145	2.67	HOXA5	‒2.04	Apoptotic
hsa-miR-602	‒2.77	IL4R	2.02	Anti-apoptotic
hsa-miR-2861	‒2.15	IRF1	2.95	Apoptotic
hsa-miR-762	‒2.04	ITGA5	2.46	Anti-apoptotic
hsa-miR-432	‒2.04	ITGA5	2.46	Anti-apoptotic
hsa-miR-146b-3p	5.6	PHF17	‒2.07	Apoptotic/ Anti-apoptotic
hsa-miR-665	‒2.75	SOCS1	2.64	Anti-apoptotic
hsa-miR-665	‒2.75	SOCS3	3.05	Anti-apoptotic
hsa-miR-1909	‒2.77	SOCS3	3.05	Anti-apoptotic
hsa-miR-1231	‒2.63	TNFSF15	2	Apoptotic
hsa-miR-762	‒2.04	TNS4	2.14	Apoptotic
hsa-miR-21*	2.34	WEE1	‒2.39	Anti-apoptotic
B. MicroRNA gene expression with their apoptotic or anti-apoptotic target mRNA gene expression at 12h, sorted by target.				
miRNA ID	miRNA fold change 12h vs 0h	Target gene	mRNA fold Change 12h vs 0h	Apoptotic or anti apoptotic Function
hsa-miR-663	‒3.19	CNFN	6.73	Anti-apoptotic
hsa-miR-146b-5p	2.73	CTNNAL1	‒2.32	Apoptotic
hsa-miR-145	2.18	FBXO32	‒2.28	Apoptotic
hsa-miR-663	‒3.19	ID1	2.65	Apoptotic/ Anti-apoptotic
hsa-miR-602	‒3.49	ID1	2.65	Apoptotic/ Anti-apoptotic
hsa-miR-494	‒3.68	ID1	2.65	Apoptotic/ Anti-apoptotic
hsa-miR-675	‒2.59	IL32	2.03	Apoptotic
hsa-miR-663	‒3.19	IL32	2.03	Apoptotic
hsa-miR-3185	‒4.44	KCTD11	2.01	Apoptotic
hsa-miR-675	‒2.59	KRT23	3.36	Apoptotic
hsa-miR-145	2.18	MPP1	‒2.7	Anti-apoptotic
hsa-miR-675	‒2.59	MUC1	2.03	Anti-apoptotic
hsa-miR-665	‒3.03	MUC1	2.03	Anti-apoptotic
hsa-miR-146b-5p	2.73	MYBL1	‒2.74	Anti-apoptotic
hsa-miR-494	‒3.68	NCF2	4.3	Apoptotic
hsa-miR-2861	‒2.96	NCF2	4.3	Apoptotic
hsa-miR-146b-5p	2.73	NR2C2AP	‒2.04	Anti-apoptotic
hsa-miR-29b-1*	2.15	PBX1	‒2.48	Apoptotic
hsa-miR-146b-3p	6.29	PBX1	‒2.48	Apoptotic
hsa-miR-602	‒3.49	PDZK1IP1	3.61	Anti-apoptotic
hsa-miR-432	2.03	PEG10	‒3.33	Anti-apoptotic
hsa-miR-1972	2.73	PEG10	‒3.33	Anti-apoptotic
hsa-miR-145	2.18	PEG10	‒3.33	Anti-apoptotic
hsa-miR-675	‒2.59	PI3	4.06	Apoptotic
hsa-miR-494	‒3.68	PLAUR	2.76	Anti-apoptotic
hsa-miR-146b-5p	2.73	PLK2	‒2.92	Apoptotic/Anti-apoptotic
hsa-miR-665	‒3.03	S100A2	2.36	Anti-apoptotic
hsa-miR-663	‒3.19	S100A9	3.07	Apoptotic
hsa-miR-494	‒3.68	SERPINB3	18.71	Anti-apoptotic
hsa-miR-494	‒3.68	SERPINB4	15.85	Anti-apoptotic
hsa-miR-596	‒3.4	SLPI	2.58	Anti-apoptotic
hsa-miR-638	‒2.57	SOD2	2.34	Anti-apoptotic
hsa-miR-3185	‒4.44	SORT1	2.14	Apoptotic
hsa-miR-663	‒3.19	TAGLN3	2.38	Apoptotic
hsa-miR-494	‒3.68	TNFRSF9	2.16	Apoptotic
hsa-miR-1972	2.73	TP53	‒2.5	Apoptotic

**Table 5:** Negatively correlated interaction between miRNAs and genes expressed in Proteomics data.

**A. Protein expression and miRNA expression at 3h**.					
miRNA ID	miRNA fold change 3h vs 0h	Target ID	Combined IonScore	Protein fold change3h vs 0h	Apoptotic Function
hsa-miR-134	2.39	CRABP2	249	‒1.1	Apoptotic
**B. Protein expression and miRNA expression at 12h**.					
miRNA ID	miRNA fold change 12h vs 0h	Target ID	Combined IonScore	Protein fold change 12h vs 0h	Apoptotic Function
hsa-miR-149*	‒2.71	CAP1	778	1.22	Apoptotic
hsa-miR-663	‒3.19	CRABP2	249	1.04	Apoptotic
hsa-miR-494	‒3.68	PDIA3	1268	1.23	Apoptotic
hsa-miR-494	‒3.68	SERPINB3	245	1.14	Anti-apoptotic

There were a number of miRNAs that had multiple negatively correlated targets, not all of which were associated with the regulation of apoptosis. The miRNAs with the greatest numbers of negatively correlated targets included miR-134, miR-145 and miR-432 at 3h post EGF treatment and miR-494 and miR-146b-5p at 12 h (S1 Table and S2 Table).

## Discussion

### Previously known miRNAs that regulate apoptosis found in our data

It is known that miRNAs regulate individual components of multiple oncogenic pathways including the EGF pathway, that regulate various biological processes including apoptosis [39]. A number of miRNAs regulate the EGF signalling pathway, for instance MIR-145 can act as a tumour suppressor and down regulates several crucial oncogenes such as ki-RAS and v-myc, it is also down regulated in various types of cancer and has been shown to induce apoptosis [39,40]. Our data show that MIR-145 is up regulated as a consequence of EGF treatment leading to apoptosis, which is consistent with previous results. We also found that miR-145 has a number of targets at 3h and 12h post EGF treatment in both gene-expression and proteome data (see Table 4, Table 5, Fig. 2A, S1 Table).

Another miRNA with a know regulatory function of interest is miR-146b-5p, which plays an important role in regulating inflammation and when over expressed may also function to inhibit apoptosis. miR-146b-5p increases in abundance and has a number of targets (PLK2 (polo-like kinase 2), MYBL1 (v-myb myeloblastosis viral oncogene homolog (avian)-like 1), NR2C2AP (nuclear receptor 2C2-associated protein), CTNNAL1 (catenin (cadherin-associated protein), alpha-like 1), DLK2 (delta-like 2 homolog (Drosophila), KIF24 (kinesin family member 24), MPHOSPH9 (M-phase phosphoprotein 9) and PER3 (period circadian clock 3) identified in our gene expression data that decrease in abundance at 3 and 12 h post EGF treatment. Of these, MYBL1 and NR2C2AP are anti-apoptotic, CTNNAL1 is pro-apoptotic and PLK2 has been reported as either pro or anti-apoptotic. So in our context, MIR-146b-5p may be acting to stimulate apoptosis.

Another miRNA of interest is miR-499–5p that has previously been shown to regulate FOXO4, which is a forkhead box O transcription factor, which can induce cell cycle arrest, DNA repair and apoptosis [41,42]. Our results show miR-499–5p to be overexpressed at 3h post EGF treatment and then down regulated by 12h post EGF. This down regulation of miR-499–5p indicates that FOXO4 may be implicated in the induction of apoptosis in our system, or it may mean that miR-499 5p has other targets that can induce apoptosis that are relevant in our context, such as BTG1, GJB6 (gap junction protein, beta 6, 30kDa), TAGLN3 (transgelin 3), TNFSF15 (tumor necrosis factor (ligand) superfamily, member 15), APOBEC3G (apolipoprotein B mRNA editing enzyme, catalytic polypeptide-like 3G) and BHLHE41 (basic helix-loop-helix family, member e41) which had negatively correlated expression (S1 Table, Table 4A).

Perhaps of particular interest is miR-494, which has previously been shown to inhibit poly (ADP-ribose) polymerase PARP cleavage, the late event of apoptosis [43]. We observed a decrease in miR-494 abundance, which was correlated with increased expression of the following pro-apoptotic genes ID1, NCF2, TNFRSF9 (tumor necrosis factor receptor superfamily, member 9), and the pro-apoptotic protein PDIA3 (protein disulfide isomerase family A, member 3) (activates caspase3). Decrease in miR-494 expression was also correlated with increased expression of anti-apoptotic genes PLAUR (plasminogen activator, urokinase receptor), SERPINB3 (serpin peptidase inhibitor, clade B (ovalbumin), member 3) (both mRNA and protein) and SERPINB4 (serpin peptidase inhibitor, clade B (ovalbumin), member 3). Because ID1, NCF2, PDIA3, PLAUR and SERPINB3 are all known to be upregulated by the interferon/JAK/STAT pathway this suggests that miR-494 was either also downregulated by JAK/STAT as another regulatory loop within this context or was regulated independently to potentiate JAK/STAT activation. This is a novel hypothesis and is thus a good choice for further investigation with respect to the regulation of apoptosis in cancer cells.

Finally, miR-663 decreases in abundance at 3h and 12 h post EGF treatment and has previously been shown to mediate chemo-resistance of breast cancer cells by suppressing the apoptotic pathway [44]. Our data support this function of MIR-663, as its down regulation coincides with the up-regulation of the following pro-apoptotic genes (ID1, IL32, S100A9 (S100 calcium binding protein A9), CRABP2 (cellular retinoic acid binding protein 2) and TAGLN3). The regulation of ID1 and IL32 reinforces the prominence of the JAK/STAT signalling pathway in this context and miR-663 is therefore also a good candidate for further investigation in this context.

### MiRNAs in our data that are novel candidates for the regulation of apoptosis

In addition to the above miRNAs that we previously been shown to regulate apoptosis, we have identified another 11 miRNAs that are good candidates for a regulatory role in apoptosis. miR-146b-3p which is up regulated, has not previously been shown to induce apoptosis but it has a number of targets at 3h and 12h post EGF treatment, some of which are known to regulate apoptosis (Fig. 2, Table 4 and S2 Table).

Another novel miRNA in this context is miR-146b-5p, also up regulated, which is known to play an important role in regulating inflammation and when over expressed may also function to inhibit apoptosis. We have found that miR-146b-5p increased in abundance and had a number of targets (PLK2, MYBL1, NR2C2AP, CTNNAL1, DLK2, KIF24, MPHOSPH9 and PER3) identified in our gene expression data that decreased in abundance at 3h and 12h post EGF treatment. Of these, MYBL1 and NR2C2AP are anti-apoptotic, CTNNAL1 is pro-apoptotic and PLK2 have been reported as either pro or anti-apoptotic. So in our context, MIR-146b-5p may be acting to stimulate apoptosis.

Another novel miRNA of interest is miR-596, which decreased along with a correlated increase in its target, BTG1, known to promote apoptosis.

Another miRNA, miR-1231 that decreased in expression is also known to target BTG1 and in addition the pro-apoptotic gene TNFSF15, both of which increased in expression.

Another novel miRNA, miR-762 which was down regulated at 3h post EGF treatment targets the up-regulated apoptotic gene TNS4 (tensin 4) which is cleaved by Caspase 3 and is also known to bind to ACTB, reinforcing the role (see below) of ACTB and its regulators in apoptosis.

Regulation of the interferon/JAK/STAT pathway was also likely via miR-2861 which was down regulated and was correlated with an increase in the expression IRF1 at 3h post EGF and network NCF2 at 12 h post EGF. This strengthens the case for a miRNA regulatory loop that was down regulated in conjunction with activation of the interferon signalling pathways.

Another miRNA without previous connection to apoptosis is miR-3185 that was down regulated and correlated with up regulation of apoptotic genes ARHGEF2 (Rho/Rac guanine nucleotide exchange factor (GEF) 2) of the interferon pathway and DCUN1D3 (DCN1, defective in cullin neddylation 1, domain containing 3 (S. cerevisiae)) at 3h post EGF treatment. It was also down regulated at 12 h post EGF and correlated with increased expression of the apoptotic genes KCTD11 (potassium channel tetramerisation domain containing 11) and SORT1.

The miRNA miR-675 decreased in abundance and was correlated with an increase in the pro-apoptotic genes IL32 (regulated by the interferon pathway), KRT23 (keratin 23) and PI3 (peptidase inhibitor 3) at 12 h post EGF treatment.

Another down regulated miRNA, miR-602 was also correlated with the increased expression of the interferon pathway regulated ID1 at 12h post EGF treatment.

In addition, miR-638 also down regulated at 12h post EGF treatment, targets the pro-apoptotic gene SOD2 (superoxide dismutase 2, mitochondrial), which was up regulated.

Finally, miR-1972, which was up regulated, targets PEG10, which normally inhibits apoptosis (also targeted by miR-432 and miR-145) and the pro-apoptotic mutant TP53 at 12 hrs. Of the eleven novel candidate miRNAs that could regulate apoptosis, 9 were down regulated, leading to apparent de-repression of primarily pro-apoptotic targets and 2 were up regulated, leading to apparent suppression of anti-apoptotic targets.

### Overlap between gene-expression and proteome miRNA targets

We have previously shown that there was little overlap between gene-expression and proteome data for A431 cells treated with EGF [18], with only 3 proteins matching regulated transcripts, and none of those present in the correlation sub-networks. However, based on miRNAs, gene-expression and proteome networks may have more in common. We found 5 miRNAs (miR-29b-1star, miR-134, miR-145, miR-432 and miR-1909) that had targets in both the gene expression and proteomic sub-networks. As mentioned above, miR-145 is known to induce apoptosis and in the proteome data it targets ACTB as does miR-29b-1star (Fig. 3, Table 6). This is of particular interest because it is known that actin cytoskeleton changes can trigger apoptosis [45]. miR-134 targets both MYB (gene expression) and BANF1 (proteome) but only MYB is known to regulate apoptosis (anti-apoptotic). However BANF1 is part of a sub-network that includes two proteins known to regulate apoptosis. miR-432 was the only miRNA that decreased at 3h and increased at 12h post EGF treatment and its target TCP1 also changed direction of gene expression at those times. TCP1, whilst not known to regulate apoptosis directly, is known to regulate actin and tubulin folding and is part of the same network as those two proteins (Fig. 3). This also supports a role for miRNA regulation of cytoskeleton integrity and is consistent with promotion of apoptosis. The potential function of miR-1909 is unclear; even though it has targets at both 3h and 12h post EGF treatment in both the gene expression and proteome data sets, the direction of change in expression and annotation of the targets do not necessarily support a role for this miRNA as a regulator of apoptosis.

**Table 6:** MicroRNAs Present In The Networks And Their Targets.

**A. MicroRNA expression and target mRNA gene expression.**							
**miRNA ID**	**miRNA fold change**		**Target gene**	**mRNA fold Change**		**Apoptotic or anti-apoptotic Function**	
	3h vs 0h	12h vs 0h		3h vs 0h	12h vs 0h		
hsa-miR-29b-1*	3.75	2.15	CFLAR	2.46	1.34	Anti-apoptotic	
hsa-miR-145	2.67	2.18	CITED2	‒2.21	1.06	Anti-apoptotic	
hsa-miR-29b-1*	3.75	2.15	EDNRA	2.16	1.85	Anti-apoptotic	
hsa-miR-432	‒2.04	2.03	ITGA5	2.46	1.66	Anti-apoptotic	
hsa-miR-145	2.67	2.18	MPP1	‒1.33	‒2.7	Anti-apoptotic	
hsa-miR-145	2.67	2.18	MUC1	1.88	2.03	Anti-apoptotic	
hsa-miR-134	2.39	1.77	MYB	‒1.91	‒2.23	Anti-apoptotic	
hsa-miR-29b-1*	3.75	2.15	NUAK2	2.59	1.28	Anti-apoptotic	
hsa-miR-145	2.67	2.18	PDZK1IP1	1.48	3.61	Anti-apoptotic	
hsa-miR-134	2.39	1.77	PDZK1IP1	1.48	3.61	Anti-apoptotic	
hsa-miR-145	2.67	2.18	PEG10	‒1.26	‒3.33	Anti-apoptotic	
hsa-miR-432	‒2.04	2.03	PEG10	‒1.26	‒3.33	Anti-apoptotic	
hsa-miR-432	‒2.04	2.03	S100A2	1.71	2.36	Anti-apoptotic	
hsa-miR-1909	‒2.77	‒5.32	SOCS3	3.05	1.55	Anti-apoptotic	
hsa-miR-134	2.39	1.77	SOD2	2.55	2.34	Anti-apoptotic	
hsa-miR-145	2.67	2.18	BTG1	2.09	1.43	Apoptotic	
hsa-miR-145	2.67	2.18	FBXO32	‒2.77	‒2.28	Apoptotic	
hsa-miR-145	2.67	2.18	HOXA5	‒2.04	‒1.65	Apoptotic	
hsa-miR-432	‒2.04	2.03	HOXA5	‒2.04	‒1.65	Apoptotic	
hsa-miR-134	2.39	1.77	ID1	1.96	2.65	Apoptotic	
hsa-miR-134	2.39	1.77	IL13RA2	1.19	2.02	Apoptotic	
hsa-miR-29b-1*	3.75	2.15	PBX1	‒1.26	‒2.48	Apoptotic	
hsa-miR-432	‒2.04	2.03	S100A9	1.42	3.07	Apoptotic	
hsa-miR-432	‒2.04	2.03	SORT1	1.38	2.14	Apoptotic	
hsa-miR-134	2.39	1.77	TAGLN3	3.15	2.38	Apoptotic	
hsa-miR-432	‒2.04	2.03	APOBEC3G	2.09	1.36	Other function	
hsa-miR-432	‒2.04	2.03	ARL5B	2.36	1.34	Other function	
hsa-miR-145	2.67	2.18	ARL5B	2.36	1.34	Other function	
hsa-miR-432	‒2.04	2.03	ARSI	‒1.42	‒2.24	Other function	
hsa-miR-432	‒2.04	2.03	BCAM	1.01	‒2.44	Other function	
hsa-miR-432	‒2.04	2.03	C1orf74	2.49	1.6	Other function	
hsa-miR-432	‒2.04	2.03	CBLB	‒2.15	‒1.54	Other function	
hsa-miR-145	2.67	2.18	CCL20	2.3	1.21	Other function	
hsa-miR-432	‒2.04	2.03	DHRS9	1.64	2.73	Other function	
hsa-miR-134	2.39	1.77	DHRS9	1.64	2.73	Other function	
hsa-miR-145	2.67	2.18	FLNB	1.5	2.08	Other function	
hsa-miR-432	‒2.04	2.03	GK	1.19	2.53	Other function	
hsa-miR-145	2.67	2.18	GRB7	2.27	1.77	Other function	
hsa-miR-29b-1*	3.75	2.15	GRHL1	2.75	3.19	Other function	
hsa-miR-145	2.67	2.18	HSD17B2	1.16	2.4	Other function	
hsa-miR-145	2.67	2.18	ITGB8	2.33	1.45	Other function	
hsa-miR-134	2.39	1.77	ITGB8	2.33	1.45	Other function	
hsa-miR-145	2.67	2.18	JHDM1D	7.01	4.71	Other function	
hsa-miR-432	‒2.04	2.03	JHDM1D	7.01	4.71	Other function	
hsa-miR-432	‒2.04	2.03	MACC1	2.9	1.17	Other function	
hsa-miR-29b-1*	3.75	2.15	MACC1	2.9	1.17	Other function	
hsa-miR-432	‒2.04	2.03	MAML2	‒1.38	‒2.19	Other function	
hsa-miR-145	2.67	2.18	MFSD2A	2.66	2.2	Other function	
hsa-miR-29b-1*	3.75	2.15	MPPED2	‒1.59	‒3.8	Other function	
hsa-miR-134	2.39	1.77	MRM1	‒1.63	‒2.02	Other function	
hsa-miR-432	2.04	2.03	MXRA5	‒1.25	‒2.16	Other function	
hsa-miR-145	2.67	2.18	NAV3	1.58	2.1	Other function	
hsa-miR-145	2.67	2.18	NFIB	‒1.19	‒2.07	Other function	
hsa-miR-1909	‒2.77	‒5.32	NFIB	‒1.19	‒2.07	Other function	
hsa-miR-145	2.67	2.18	NLRC5	2.52	1.17	Other function	
hsa-miR-432	‒2.04	2.03	NRM	‒1.2	‒2.3	Other function	
hsa-miR-145	2.67	2.18	NTN4	‒1.32	‒2.08	Other function	
hsa-miR-145	2.67	2.18	PLCE1	‒1.22	‒2.15	Other function	
hsa-miR-432	‒2.04	2.03	PRSS22	2.82	1.95	Other function	
hsa-miR-1909	‒2.77	‒5.32	RAB43	2.67	1.5	Other function	
hsa-miR-134	2.39	1.77	RHCG	1.1	2.32	Other function	
hsa-miR-134	2.39	1.77	SDCBP2	1.47	2.75	Other function	
hsa-miR-1909	‒2.77	‒5.32	SEMA7A	3.23	1.16	Other function	
hsa-miR-134	2.39	1.77	SPRR2A	6.33	6.25	Other function	
hsa-miR-145	2.67	2.18	SQRDL	1.56	2.69	Other function	
hsa-miR-134	2.39	1.77	SQRDL	1.56	2.69	Other function	
hsa-miR-145	2.67	2.18	SRGAP1	‒2.19	‒1.58	Other function	
hsa-miR-432	‒2.04	2.03	THNSL1	‒2.31	‒1.67	Other function	
**B. MicroRNA expression and Protein (target) expression**.							
**miRNA ID**	**miRNA fold change**		**Target ID**	**Combined IonScore**	**Protein fold change**		**Apoptotic or anti-apoptotic Function**	
	**3h vs 0h**	**12h vs 0h**			**3h vs 0h**	**12h vs 0h**		
hsa-miR-1909	‒2.77	‒5.32	CFL1	984	‒1.27	‒1.42	Apoptotic
hsa-miR-134	2.39	1.77	CRABP2	249	‒1.1	1.04	Apoptotic
hsa-miR-145	2.67	2.18	ACTB	357	‒1.06	‒1.13	Other function
hsa-miR-29b-1*	3.75	2.15	ACTB	357	‒1.06	‒1.13	Other function
hsa-miR-134	2.39	1.77	BANF1	158	‒1.09	‒1.15	Other function
hsa-miR-432	‒2.04	2.03	TCP1	2763	‒1.04	1.14	Other function

## Conclusions

We have confirmed roles for some miRNAs in EGF signalling and apoptosis and implicated some miRNAs that previously had not been associated with EGF signalling or apoptosis. Our results suggest that miRNAs that target pro-apoptotic genes tend to decrease in abundance while their targets increase and that other miRNAs that target anti-apoptotic genes increase in abundance while their targets decrease. Eight miRNAs (miR-494, miR-499–5p, miR-602, miR-663, miR-675, miR-1231, miR-2861 and miR-3185) that could normally target interferon pathway induced pro-apoptotic transcripts were found to be down regulated, leading us to conclude that miRNAs may provide a novel layer of regulation within the interferon pathway, specifically with respect to components downstream of JAK/STAT. Finally, we speculate that a pull/push mechanism exists to favour the expression of pro-apoptotic genes as a result of high dose EGF treatment.

## Supporting Information

S1 Table Differentially regulated interaction between miRNAs and genes in gene expression data in A431 cells after EGF treatment. (DOCX)

S2 Table Differentially regulated interaction between miRNAs and genes in Proteomics data in A431 cells after EGF treatment. (DOCX)

## References

[1] Normanno N, Bianco C, Strizzi L, Mancino M, Maiello MR, De Luca A, et al (2005) The ErbB receptors and their ligands in cancer: An overview. Current Drug Targets 6: 243–257. 15857286 10.2174/1389450053765879

[2] Olayioye MA, Neve RM, Lane HA, Hynes NE (2000) The ErbB signaling network: receptor heterodimerization in development and cancer. Embo Journal 19: 3159–3167. 10880430 10.1093/emboj/19.13.3159 PMC313958

[3] Rodeck U, Jost M, Kari C, Shih DT, Lavker RM, Ewert DL, et al (1997) EGF-R dependent regulation of keratinocyte survival. J Cell Sci 110 (Pt 2): 113–121. 9044042 10.1242/jcs.110.2.113

[4] Kari C, Chan TO, Rocha De Quadros M, Rodeck U (2003) Targeting the epidermal growth factor receptor in cancer: apoptosis takes center stage. Cancer Res 63: 1–5. 12517767

[5] Schneeweiss A, Kolay S, Aulmann S, Von Minckwitz G, Torode J, Koehler M, et al (2004) Induction of remission in a patient with metastatic breast cancer refractory to trastuzumab and chemotherapy following treatment with gefitinib ('Iressa', ZD1839). Anticancer Drugs 15: 235–238. 15014356 10.1097/00001813-200403000-00007

[6] Ciardiello F, Tortora G (2001) A novel approach in the treatment of cancer: Targeting the epidermal growth factor receptor. Clinical Cancer Research 7: 2958–2970. 11595683

[7] Janmaat ML, Giaccone G (2003) The epidermal growth factor receptor pathway and its inhibition as anticancer therapy. Drugs Today (Barc) 39 Suppl C: 61–80. 14988746

[8] Solomon B, Hagekyriakou J, Trivett MK, Stacker SA, McArthur GA, Cullinane C (2003) EGFR blockade with ZD1839 ("Iressa") potentiates the antitumor effects of single and multiple fractions of ionizing radiation in human A431 squamous cell carcinoma. International Journal of Radiation Oncology Biology Physics 55: 713–723. 10.1016/s0360-3016(02)04357-2 12573759

[9] Schmidt M, Lichtner RB (2002) EGF receptor targeting in therapy-resistant human tumors. Drug Resistance Updates 5: 11–18. 12127860 10.1016/s1368-7646(02)00004-3

[10] Woodburn JR (1999) The epidermal growth factor receptor and its inhibition in cancer therapy. Pharmacol Ther 82: 241–250. 10454201 10.1016/s0163-7258(98)00045-x

[11] Gill GN, Lazar CS (1981) Increased phosphotyrosine content and inhibition of proliferation in EGF-treated A431 cells. Nature 293: 305–307. 6268987 10.1038/293305a0

[12] Barnes DW (1982) Epidermal Growth-Factor Inhibits Growth of A431 Human Epidermoid Carcinoma in Serum-Free Cell-Culture. Journal of Cell Biology 93: 1–4. 7040412 10.1083/jcb.93.1.1 PMC2112100

[13] Gill GN, Buss JE, Lazar CS, Lifshitz A, Cooper JA (1982) Role of epidermal growth factor-stimulated protein kinase in control of proliferation of A431 cells. J Cell Biochem 19: 249–257. 6296169 10.1002/jcb.240190306

[14] Gulli LF, Palmer KC, Chen YQ, Reddy KB (1996) Epidermal growth factor-induced apoptosis in A431 cells can be reversed by reducing the tyrosine kinase activity. Cell Growth Differ 7: 173–178. 8822200

[15] Weinstein EJ, Grimm S, Leder P (1998) The oncogene heregulin induces apoptosis in breast epithelial cells and tumors. Oncogene 17: 2107–2113. 9798682 10.1038/sj.onc.1202428

[16] Armstrong DK, Kaufmann SH, Ottaviano YL, Furuya Y, Buckley JA, Isaacs JT, et al (1994) Epidermal growth factor-mediated apoptosis of MDA-MB-468 human breast cancer cells. Cancer Res 54: 5280–5283. 7923154

[17] Pinkas-Kramarski R, Alroy I, Yarden Y (1997) ErbB receptors and EGF-like ligands: cell lineage determination and oncogenesis through combinatorial signaling. J Mammary Gland Biol Neoplasia 2: 97–107. 10882296 10.1023/a:1026343528967

[18] Alanazi I, Ebrahimie E, Hoffmann P, Adelson DL (2013) Combined gene expression and proteomic analysis of EGF induced apoptosis in A431 cells suggests multiple pathways trigger apoptosis. Apoptosis: an international journal on programmed cell death. 10.1007/s10495-013-0887-6 23892916

[19] Lin C-C, Chen Y-J, Chen C-Y, Oyang Y-J, Juan H-F, Huang H-C (2012) Crosstalk between transcription factors and microRNAs in human protein interaction network. BMC Systems Biology 6: 18 10.1186/1752-0509-6-18 22413876 PMC3337275

[20] Qiu CX, Wang JA, Yao PY, Wang E, Cui QH (2010) microRNA evolution in a human transcription factor and microRNA regulatory network. BMC Systems Biology 4. 10.1186/1752-0509-4-90 PMC2914650 20584335

[21] Shi M, Guo N (2009) MicroRNA expression and its implications for the diagnosis and therapeutic strategies of breast cancer. Cancer treatment reviews 35: 328–334. 10.1016/j.ctrv.2008.12.002 19171434

[22] Iorio MV, Ferracin M, Liu CG, Veronese A, Spizzo R, Sabbioni S, et al (2005) MicroRNA gene expression deregulation in human breast cancer. Cancer Research 65: 7065–7070. 16103053 10.1158/0008-5472.CAN-05-1783

[23] Rossi S, Sevignani C, Nnadi SC, Siracusa LD, Calin GA (2008) Cancer-associated genomic regions (CAGRs) and noncoding RNAs: bioinformatics and therapeutic implications. Mammalian genome: official journal of the International Mammalian Genome Society 19: 526–540. 10.1007/s00335-008-9119-8 18636290

[24] Cerchia L, De Franciscis V (2006) Noncoding RNAs in cancer medicine. Journal of biomedicine & biotechnology 2006: 73104. 17057370 10.1155/JBB/2006/73104 PMC1559931

[25] Bartel DP (2004) MicroRNAs: genomics, biogenesis, mechanism, and function. Cell 116: 281–297. 14744438 10.1016/s0092-8674(04)00045-5

[26] Flynt AS, Lai EC (2008) Biological principles of microRNA-mediated regulation: shared themes amid diversity. Nature reviews Genetics 9: 831–842. 10.1038/nrg2455 18852696 PMC2729318

[27] Kim VN, Nam JW (2006) Genomics of microRNA. Trends in genetics: TIG 22: 165–173. 16446010 10.1016/j.tig.2006.01.003

[28] Lima RT, Busacca S, Almeida GM, Gaudino G, Fennell DA, Vasconcelos MH (2011) MicroRNA regulation of core apoptosis pathways in cancer. European Journal of Cancer 47: 163–174. 10.1016/j.ejca.2010.11.005 21145728

[29] Gentleman RC, Carey VJ, Bates DM, Bolstad B, Dettling M, Dudoit S, et al (2004) Bioconductor: open software development for computational biology and bioinformatics. Genome Biology 5: R80 15461798 10.1186/gb-2004-5-10-r80 PMC545600

[30] Bolstad BM, Irizarry RA, Astrand M, Speed TP (2003) A comparison of normalization methods for high density oligonucleotide array data based on variance and bias. Bioinformatics 19: 185–193. 12538238 10.1093/bioinformatics/19.2.185

[31] Wettenhall JM, Smyth GK (2004) limmaGUI: a graphical user interface for linear modeling of microarray data. Bioinformatics 20: 3705–3706. 15297296 10.1093/bioinformatics/bth449

[32] Wu G, Stein L (2012) A network module-based method for identifying cancer prognostic signatures. Genome Biology 13: R112 10.1186/gb-2012-13-12-r112 23228031 PMC3580410

[33] Thomas PD, Campbell MJ, Kejariwal A, Mi H, Karlak B, Daverman R, et al (2003) PANTHER: a library of protein families and subfamilies indexed by function. Genome research 13: 2129–2141. 12952881 10.1101/gr.772403 PMC403709

[34] Liu H, Takeuchi S, Moroi Y, Lin N, Urabe K, Kokuba H, et al (2007) Expression of minichromosome maintenance 5 protein in proliferative and malignant skin diseases. International journal of dermatology 46: 1171–1176. 17988337 10.1111/j.1365-4632.2007.03335.x

[35] Cande C, Vahsen N, Kouranti I, Schmitt E, Daugas E, Spahr C, et al (2004) AIF and cyclophilin A cooperate in apoptosis-associated chromatinolysis. Oncogene 23: 1514–1521. 14716299 10.1038/sj.onc.1207279

[36] Walsh B, Pearl A, Suchy S, Tartaglio J, Visco K, Phelan SA (2009) Overexpression of Prdx6 and resistance to peroxide-induced death in Hepa1–6 cells: Prdx suppression increases apoptosis. Redox report: communications in free radical research 14: 275–284. 10.1179/135100009X12525712409652 20003713

[37] Nahta R, Yuan LX, Fiterman DJ, Zhang L, Symmans WF, Ueno NT, et al (2006) B cell translocation gene 1 contributes to antisense Bcl-2-mediated apoptosis in breast cancer cells. Molecular Cancer Therapeutics 5: 1593–1601. 16818519 10.1158/1535-7163.MCT-06-0133

[38] Sun XH, Yang YZ, Wang HC (2008) IdI induces apoptosis through inhibition of RORgammat expression. Bmc Immunology 9. 10.1186/1471-2172-9-20 PMC2408562 18489764

[39] Mlcochova J, Faltejskova P, Nemecek R, Svoboda M, Slaby O (2013) MicroRNAs targeting EGFR signalling pathway in colorectal cancer. Journal of cancer research and clinical oncology. 10.1007/s00432-013-1470-9 PMC11824681 23817698

[40] Zhang J, Sun Q, Zhang Z, Ge S, Han ZG, Chen WT (2013) Loss of microRNA-143/145 disturbs cellular growth and apoptosis of human epithelial cancers by impairing the MDM2-p53 feedback loop. Oncogene 32: 61–69. 10.1038/onc.2012.28 22330136

[41] Greer EL, Brunet A (2005) FOXO transcription factors at the interface between longevity and tumor suppression. Oncogene 24: 7410–7425. 16288288 10.1038/sj.onc.1209086

[42] Liu XQ, Zhang ZY, Sun L, Chai N, Tang SH, Jin J, et al (2011) microRNA-499–5p promotes cellular invasion and tumor metastasis in colorectal cancer by targeting FOXO4 and PDCD4. Carcinogenesis 32: 1798–1805. 10.1093/carcin/bgr213 21934092

[43] Romano G, Acunzo M, Garofalo M, Di Leva G, Cascione L, Zanca C, et al (2012) MiR-494 is regulated by ERK1/2 and modulates TRAIL-induced apoptosis in non-small-cell lung cancer through BIM down-regulation. Proceedings of the National Academy of Sciences of the United States of America 109: 16570–16575. 10.1073/pnas.1207917109 23012423 PMC3478630

[44] Hu H, Li S, Cui X, Lv X, Jiao Y, Yu F, et al (2013) The overexpression of hypomethylated miR-663 induces chemotherapy resistance in human breast cancer cells by targeting heparin sulfate proteoglycan 2 (HSPG2). The Journal of biological chemistry 288: 10973–10985. 10.1074/jbc.M112.434340 23436656 PMC3630848

[45] Papadopoulou N, Charalampopoulos I, Alevizopoulos K, Gravanis A, Stournaras C (2008) Rho/ROCK/actin signaling regulates membrane androgen receptor induced apoptosis in prostate cancer cells. Experimental Cell Research 314: 3162–3174. 10.1016/j.yexcr.2008.07.012 18694745

