# Supplementary material for: MicroRNAs Are Part of the Regulatory Network that Controls EGF Induced Apoptosis, Including Elements of the JAK/STAT Pathway, in A431 Cells
Source: PLoS One. 2015 Mar 17;10(3):e0120337. doi: 10.1371/journal.pone.0120337 (PMC4364457; doi:10.1371/journal.pone.0120337)
Supplement: S1 Table — (DOCX) [file pone.0120337.s001.docx]

Supplementary Table 1: Differentially regulated interaction between miRNAs and genes in gene expression data in A431 cells after EGF treatment.

A- MicroRNA gene expression with their target mRNA gene expression at 3h.

| miRNA ID | miRNA fold change 3h vs 0h | Target gene | mRNA fold Change 3h vs 0h | Apoptotic or anti-apoptotic Function |
| --- | --- | --- | --- | --- |
| hsa-miR-92a-1* | 4.39 | CFLAR | 2.46 | Anti-apoptotic |
| hsa-miR-762 | -2.04 | ITGA5 | 2.46 | Anti-apoptotic |
| hsa-miR-762 | -2.04 | TNS4 | 2.14 | Apoptotic |
| hsa-miR-762 | -2.04 | RAB43 | 2.67 | Other function |
| hsa-miR-675 | -2.27 | JUNB | 2.08 | Other function |
| hsa-miR-665 | -2.75 | SOCS1 | 2.64 | Anti-apoptotic |
| hsa-miR-665 | -2.75 | SOCS3 | 3.05 | Anti-apoptotic |
| hsa-miR-665 | -2.75 | BCL3 | 3.81 | Anti-apoptotic |
| hsa-miR-665 | -2.75 | OVOL1 | 4.72 | Other function |
| hsa-miR-663 | -1.85 | TAGLN3 | 3.15 | Apoptotic |
| hsa-miR-663 | -1.85 | ARL4C | -2.16 | Other function |
| hsa-miR-663 | -1.85 | JHDM1D | 7.01 | Other function |
| hsa-miR-663 | -1.85 | JUNB | 2.08 | Other function |
| hsa-miR-663 | -1.85 | MFSD2A | 2.66 | Other function |
| hsa-miR-663 | -1.85 | PRSS22 | 2.82 | Other function |
| hsa-miR-663 | -1.85 | RASSF3 | 2.13 | Other function |
| hsa-miR-638 | -1.94 | SOD2 | 2.55 | Anti-apoptotic |
| hsa-miR-638 | -1.94 | GDF15 | 2.19 | Apoptotic |
| hsa-miR-638 | -1.94 | HIST1H4C | 2.47 | Other function |
| hsa-miR-638 | -1.94 | HIST2H4A | -2.14 | Other function |
| hsa-miR-638 | -1.94 | SPRR2A | 6.33 | Other function |
| hsa-miR-602 | -2.77 | IL4R | 2.02 | Anti-apoptotic |
| hsa-miR-602 | -2.77 | RAB43 | 2.67 | Other function |
| hsa-miR-602 | -2.77 | SPRR2D | 2.29 | Other function |
| hsa-miR-596 | -2.6 | BTG1 | 2.09 | Apoptotic |
| hsa-miR-596 | -2.6 | S100A3 | 2.04 | Other function |
| hsa-miR-596 | -2.6 | SECTM1 | 2.67 | Other function |
| hsa-miR-499-5p | 2.26 | BHLHE41 | -2.37 | Anti-apoptotic |
| hsa-miR-499-5p | 2.26 | PIM1 | 2.02 | Anti-apoptotic |
| hsa-miR-499-5p | 2.26 | CDKN1A | 4.24 | Anti-apoptotic |
| hsa-miR-499-5p | 2.26 | BTG1 | 2.09 | Apoptotic |
| hsa-miR-499-5p | 2.26 | GJB6 | 2.03 | Apoptotic |
| hsa-miR-499-5p | 2.26 | TAGLN3 | 3.15 | Apoptotic |
| hsa-miR-499-5p | 2.26 | TNFSF15 | 2 | Apoptotic |
| hsa-miR-499-5p | 2.26 | APOBEC3G | 2.09 | Other function |
| hsa-miR-499-5p | 2.26 | HAS2 | 10.69 | Other function |
| hsa-miR-499-5p | 2.26 | HIST1H4C | 2.47 | Other function |
| hsa-miR-499-5p | 2.26 | HIST2H4A | -2.14 | Other function |
| hsa-miR-499-5p | 2.26 | JHDM1D | 7.01 | Other function |
| hsa-miR-499-5p | 2.26 | MAST4 | 2.09 | Other function |
| hsa-miR-499-5p | 2.26 | RTKN2 | 2.71 | Other function |
| hsa-miR-499-5p | 2.26 | TRERF1 | -2.04 | Other function |
| hsa-miR-494 | -1.38 | SERPINB4 | 3.62 | Anti-apoptotic |
| hsa-miR-494 | -1.38 | SERPINB3 | 4.11 | Anti-apoptotic |
| hsa-miR-494 | -1.38 | PLAUR | 5.13 | Anti-apoptotic |
| hsa-miR-494 | -1.38 | EFNB2 | 2 | Apoptotic |
| hsa-miR-494 | -1.38 | LIF | 9.87 | Apoptotic |
| hsa-miR-494 | -1.38 | MAP3K8 | 2.19 | Apoptotic |
| hsa-miR-494 | -1.38 | OCLN | 2.85 | Apoptotic |
| hsa-miR-494 | -1.38 | PTPN12 | 2.06 | Apoptotic |
| hsa-miR-494 | -1.38 | SGMS2 | 2.27 | Apoptotic |
| hsa-miR-494 | -1.38 | TNFRSF9 | 2.43 | Apoptotic |
| hsa-miR-494 | -1.38 | TNFSF15 | 2 | Apoptotic |
| hsa-miR-494 | -1.38 | GRHL1 | 2.75 | Other function |
| hsa-miR-494 | -1.38 | IL1R2 | 2.26 | Other function |
| hsa-miR-494 | -1.38 | MACC1 | 2.9 | Other function |
| hsa-miR-494 | -1.38 | PTPRE | 2.08 | Other function |
| hsa-miR-494 | -1.38 | RTKN2 | 2.71 | Other function |
| hsa-miR-494 | -1.38 | TAP1 | 2.04 | Other function |
| hsa-miR-494 | -1.38 | TRERF1 | -2.04 | Other function |
| hsa-miR-494 | -1.38 | ZNF750 | 5.77 | Other function |
| hsa-miR-432 | -2.04 | ITGA5 | 2.46 | Anti-apoptotic |
| hsa-miR-432 | -2.04 | HOXA5 | -2.04 | Apoptotic |
| hsa-miR-432 | -2.04 | APOBEC3G | 2.09 | Other function |
| hsa-miR-432 | -2.04 | ARL5B | 2.36 | Other function |
| hsa-miR-432 | -2.04 | C1orf74 | 2.49 | Other function |
| hsa-miR-432 | -2.04 | CBLB | -2.15 | Other function |
| hsa-miR-432 | -2.04 | JHDM1D | 7.01 | Other function |
| hsa-miR-432 | -2.04 | MACC1 | 2.9 | Other function |
| hsa-miR-432 | -2.04 | PRSS22 | 2.82 | Other function |
| hsa-miR-432 | -2.04 | THNSL1 | -2.31 | Other function |
| hsa-miR-3185 | -2.9 | ARHGEF2 | 2.42 | Apoptotic |
| hsa-miR-3185 | -2.9 | DCUN1D3 | 3.08 | Apoptotic |
| hsa-miR-3185 | -2.9 | ARL4C | -2.16 | Other function |
| hsa-miR-3185 | -2.9 | JHDM1D | 7.01 | Other function |
| hsa-miR-3185 | -2.9 | SRGAP1 | -2.19 | Other function |
| hsa-miR-29b-1* | 3.75 | EDNRA | 2.16 | Anti-apoptotic |
| hsa-miR-29b-1* | 3.75 | CFLAR | 2.46 | Anti-apoptotic |
| hsa-miR-29b-1* | 3.75 | NUAK2 | 2.59 | Anti-apoptotic |
| hsa-miR-29b-1* | 3.75 | GRHL1 | 2.75 | Other function |
| hsa-miR-29b-1* | 3.75 | MACC1 | 2.9 | Other function |
| hsa-miR-2861 | -2.15 | IRF1 | 2.95 | Apoptotic |
| hsa-miR-2861 | -2.15 | ARL4C | -2.16 | Other function |
| hsa-miR-222* | 4.35 | CBLB | -2.15 | Other function |
| hsa-miR-21* | 2.34 | WEE1 | -2.39 | Anti-apoptotic |
| hsa-miR-21* | 2.34 | MAP3K8 | 2.19 | Apoptotic |
| hsa-miR-21* | 2.34 | SGMS2 | 2.27 | Apoptotic |
| hsa-miR-21* | 2.34 | GRHL1 | 2.75 | Other function |
| hsa-miR-21* | 2.34 | GRHL1 | 2.75 | Other function |
| hsa-miR-1972 | 1.3 | DCUN1D3 | 3.08 | Apoptotic |
| hsa-miR-1972 | 1.3 | PHF17 | -2.07 | Apoptotic/ Anti-apoptotic |
| hsa-miR-1909 | -2.77 | SOCS3 | 3.05 | Anti-apoptotic |
| hsa-miR-1909 | -2.77 | RAB43 | 2.67 | Other function |
| hsa-miR-1909 | -2.77 | SEMA7A | 3.23 | Other function |
| hsa-miR-1908 | -2 | ARL4C | -2.16 | Other function |
| hsa-miR-1908 | -2 | JUNB | 2.08 | Other function |
| hsa-miR-149* | -1.93 | JUNB | 2.08 | Other function |
| hsa-miR-149* | -1.93 | RNF19B | 2.29 | Other function |
| hsa-miR-149* | -1.93 | SEMA7A | 3.23 | Other function |
| hsa-miR-146b-5p | 1.7 | SOD2 | 2.55 | Anti-apoptotic |
| hsa-miR-146b-5p | 1.7 | PLAUR | 5.13 | Anti-apoptotic |
| hsa-miR-146b-5p | 1.7 | APOL6 | 2.37 | Apoptotic |
| hsa-miR-146b-5p | 1.7 | PLK2 | -2.45 | Apoptotic/ Anti-apoptotic |
| hsa-miR-146b-3p | 5.6 | PIM1 | 2.02 | Anti-apoptotic |
| hsa-miR-146b-3p | 5.6 | DCUN1D3 | 3.08 | Apoptotic |
| hsa-miR-146b-3p | 5.6 | PRDM1 | 9.05 | Apoptotic |
| hsa-miR-146b-3p | 5.6 | PTPN12 | 2.06 | Apoptotic |
| hsa-miR-146b-3p | 5.6 | PHF17 | -2.07 | Apoptotic/ Anti-apoptotic |
| hsa-miR-146b-3p | 5.6 | ARL4C | -2.16 | Other function |
| hsa-miR-146b-3p | 5.6 | GRB7 | 2.27 | Other function |
| hsa-miR-146b-3p | 5.6 | GRHL1 | 2.75 | Other function |
| hsa-miR-146b-3p | 5.6 | SAMD9L | 2.1 | Other function |
| hsa-miR-146b-3p | 5.6 | ZNF488 | -2.71 | Other function |
| hsa-miR-145 | 2.67 | CITED2 | -2.21 | Anti-apoptotic |
| hsa-miR-145 | 2.67 | BTG1 | 2.09 | Apoptotic |
| hsa-miR-145 | 2.67 | FBXO32 | -2.77 | Apoptotic |
| hsa-miR-145 | 2.67 | HOXA5 | -2.04 | Apoptotic |
| hsa-miR-145 | 2.67 | ARL5B | 2.36 | Other function |
| hsa-miR-145 | 2.67 | CCL20 | 2.3 | Other function |
| hsa-miR-145 | 2.67 | GRB7 | 2.27 | Other function |
| hsa-miR-145 | 2.67 | ITGB8 | 2.33 | Other function |
| hsa-miR-145 | 2.67 | JHDM1D | 7.01 | Other function |
| hsa-miR-145 | 2.67 | MFSD2A | 2.66 | Other function |
| hsa-miR-145 | 2.67 | NLRC5 | 2.52 | Other function |
| hsa-miR-145 | 2.67 | SRGAP1 | -2.19 | Other function |
| hsa-miR-134 | 2.39 | SOD2 | 2.55 | Anti-apoptotic |
| hsa-miR-134 | 2.39 | TAGLN3 | 3.15 | Apoptotic |
| hsa-miR-134 | 2.39 | ITGB8 | 2.33 | Other function |
| hsa-miR-134 | 2.39 | SPRR2A | 6.33 | Other function |
| hsa-miR-1231 | -2.63 | BTG1 | 2.09 | Apoptotic |
| hsa-miR-1231 | -2.63 | TNFSF15 | 2 | Apoptotic |

B- MicroRNA gene expression with their target mRNA gene expression at 12h.

| miRNA ID | miRNA fold change 12h vs 0h | Target gene | mRNA fold Change 12h vs 0h | Apoptotic or anti apoptotic Function |
| --- | --- | --- | --- | --- |
| hsa-miR-92a-1* | 5.55 | CBX5 | -2.2 | other function |
| hsa-miR-92a-1* | 5.55 | NRM | -2.03 | other function |
| hsa-miR-762 | -3.36 | CBX5 | -2.2 | other function |
| hsa-miR-762 | -3.36 | CCDC113 | -2.26 | other function |
| hsa-miR-762 | -3.36 | NAV2 | -2.08 | other function |
| hsa-miR-675 | -2.59 | MUC1 | 2.03 | Anti-apoptotic |
| hsa-miR-675 | -2.59 | IL32 | 2.03 | Apoptotic |
| hsa-miR-675 | -2.59 | KRT23 | 3.36 | Apoptotic |
| hsa-miR-675 | -2.59 | PI3 | 4.06 | Apoptotic |
| hsa-miR-675 | -2.59 | DLK2 | -2.02 | other function |
| hsa-miR-675 | -2.59 | NRM | -2.3 | other function |
| hsa-miR-675 | -2.59 | SCARA3 | -2.36 | other function |
| hsa-miR-665 | -3.03 | S100A2 | 2.36 | Anti-apoptotic |
| hsa-miR-665 | -3.03 | MUC1 | 2.03 | Anti-apoptotic |
| hsa-miR-665 | -3.03 | BCAM | -2.44 | other function |
| hsa-miR-665 | -3.03 | BRIP1 | -2.93 | other function |
| hsa-miR-665 | -3.03 | CSNK1G1 | -2.06 | other function |
| hsa-miR-665 | -3.03 | IL23A | 2.32 | other function |
| hsa-miR-665 | -3.03 | KRT16 | 2.43 | other function |
| hsa-miR-665 | -3.03 | KRT4 | -2.43 | other function |
| hsa-miR-665 | -3.03 | OVOL1 | 2.01 | other function |
| hsa-miR-665 | -3.03 | SLC10A6 | 3.06 | other function |
| hsa-miR-663 | -3.19 | CNFN | 6.73 | Anti-apoptotic |
| hsa-miR-663 | -3.19 | ID1 | 2.65 | Apoptotic/Anti-apoptotic |
| hsa-miR-663 | -3.19 | IL32 | 2.03 | Apoptotic |
| hsa-miR-663 | -3.19 | S100A9 | 3.07 | Apoptotic |
| hsa-miR-663 | -3.19 | TAGLN3 | 2.38 | Apoptotic |
| hsa-miR-663 | -3.19 | JHDM1D | 4.71 | other function |
| hsa-miR-663 | -3.19 | MCM5 | -2.05 | other function |
| hsa-miR-663 | -3.19 | MFSD2A | 2.2 | other function |
| hsa-miR-638 | -2.57 | SOD2 | 2.34 | Anti-apoptotic |
| hsa-miR-638 | -2.57 | BCAM | -2.44 | other function |
| hsa-miR-638 | -2.57 | SPRR2A | 6.25 | other function |
| hsa-miR-638 | -2.57 | SPRR2E | 2.09 | other function |
| hsa-miR-602 | -3.49 | PDZK1IP1 | 3.61 | Anti-apoptotic |
| hsa-miR-602 | -3.49 | ID1 | 2.65 | Apoptotic/Anti-apoptotic |
| hsa-miR-602 | -3.49 | FAM25B | 2.31 | other function |
| hsa-miR-602 | -3.49 | SPRR2E | 2.09 | other function |
| hsa-miR-596 | -3.4 | SLPI | 2.58 | Anti-apoptotic |
| hsa-miR-596 | -3.4 | PBX1 | -2.48 | Apoptotic |
| hsa-miR-596 | -3.4 | FOXRED1 | -2.07 | other function |
| hsa-miR-596 | -3.4 | NAV3 | 2.1 | other function |
| hsa-miR-596 | -3.4 | NFIB | -2.07 | other function |
| hsa-miR-596 | -3.4 | RHCG | 2.32 | other function |
| hsa-miR-596 | -3.4 | SDCBP2 | 2.75 | other function |
| hsa-miR-596 | -3.4 | SECTM1 | 3.13 | other function |
| hsa-miR-499-5p | -1.22 | MYB | -2.23 | Anti-apoptotic |
| hsa-miR-499-5p | -1.22 | HELLS | -2.38 | Anti-apoptotic |
| hsa-miR-499-5p | -1.22 | CEACAM5 | 2.48 | Anti-apoptotic |
| hsa-miR-499-5p | -1.22 | CDKN1A | 2.67 | Anti-apoptotic |
| hsa-miR-499-5p | -1.22 | EXO1 | -2.29 | Apoptotic |
| hsa-miR-499-5p | -1.22 | GJB6 | 2.37 | Apoptotic |
| hsa-miR-499-5p | -1.22 | IL13RA2 | 2.02 | Apoptotic |
| hsa-miR-499-5p | -1.22 | TAGLN3 | 2.38 | Apoptotic |
| hsa-miR-499-5p | -1.22 | CROT | -2.37 | other function |
| hsa-miR-499-5p | -1.22 | CSNK1G1 | -2.06 | other function |
| hsa-miR-499-5p | -1.22 | CXCL17 | 2.19 | other function |
| hsa-miR-499-5p | -1.22 | HAS2 | 19.09 | other function |
| hsa-miR-499-5p | -1.22 | HSD17B2 | 2.4 | other function |
| hsa-miR-499-5p | -1.22 | IL23A | 2.32 | other function |
| hsa-miR-499-5p | -1.22 | JHDM1D | 4.71 | other function |
| hsa-miR-499-5p | -1.22 | MPPED2 | -3.8 | other function |
| hsa-miR-499-5p | -1.22 | MYH10 | -2.04 | other function |
| hsa-miR-499-5p | -1.22 | NAV2 | -2.08 | other function |
| hsa-miR-499-5p | -1.22 | RAD51AP1 | -2.06 | other function |
| hsa-miR-499-5p | -1.22 | RHOBTB3 | -3.19 | other function |
| hsa-miR-499-5p | -1.22 | SPRR1B | 3.15 | other function |
| hsa-miR-494 | -3.68 | SERPINB4 | 15.85 | Anti-apoptotic |
| hsa-miR-494 | -3.68 | SERPINB3 | 18.71 | Anti-apoptotic |
| hsa-miR-494 | -3.68 | PLAUR | 2.76 | Anti-apoptotic |
| hsa-miR-494 | -3.68 | ID1 | 2.65 | Apoptotic/Anti-apoptotic |
| hsa-miR-494 | -3.68 | NCF2 | 4.3 | Apoptotic |
| hsa-miR-494 | -3.68 | TNFRSF9 | 2.16 | Apoptotic |
| hsa-miR-494 | -3.68 | AQP3 | 2.43 | other function |
| hsa-miR-494 | -3.68 | BRIP1 | -2.93 | other function |
| hsa-miR-494 | -3.68 | CSNK1G1 | -2.06 | other function |
| hsa-miR-494 | -3.68 | GK | 2.53 | other function |
| hsa-miR-494 | -3.68 | GRHL1 | 3.19 | other function |
| hsa-miR-494 | -3.68 | IL1R2 | 5.77 | other function |
| hsa-miR-494 | -3.68 | NAV2 | -2.08 | other function |
| hsa-miR-494 | -3.68 | NFIB | -2.07 | other function |
| hsa-miR-494 | -3.68 | TAP1 | 2.04 | other function |
| hsa-miR-494 | -3.68 | WDHD1 | -2.16 | other function |
| hsa-miR-494 | -3.68 | ZNF750 | 4.2 | other function |
| hsa-miR-432 | 2.03 | PEG10 | -3.33 | Anti-apoptotic |
| hsa-miR-432 | 2.03 | S100A2 | 2.36 | Anti-apoptotic |
| hsa-miR-432 | 2.03 | S100A9 | 3.07 | Apoptotic |
| hsa-miR-432 | 2.03 | SORT1 | 2.14 | Apoptotic |
| hsa-miR-432 | 2.03 | ARSI | -2.24 | other function |
| hsa-miR-432 | 2.03 | BCAM | -2.44 | other function |
| hsa-miR-432 | 2.03 | DHRS9 | 2.73 | other function |
| hsa-miR-432 | 2.03 | GK | 2.53 | other function |
| hsa-miR-432 | 2.03 | JHDM1D | 4.71 | other function |
| hsa-miR-432 | 2.03 | MAML2 | -2.19 | other function |
| hsa-miR-432 | 2.03 | MXRA5 | -2.16 | other function |
| hsa-miR-432 | 2.03 | NRM | -2.3 | other function |
| hsa-miR-3185 | -4.44 | KCTD11 | 2.01 | Apoptotic |
| hsa-miR-3185 | -4.44 | SORT1 | 2.14 | Apoptotic |
| hsa-miR-3185 | -4.44 | JHDM1D | 4.71 | other function |
| hsa-miR-29b-1* | 2.15 | PBX1 | -2.48 | Apoptotic |
| hsa-miR-29b-1* | 2.15 | GRHL1 | 3.19 | other function |
| hsa-miR-29b-1* | 2.15 | MPPED2 | -3.8 | other function |
| hsa-miR-2861 | -2.96 | NCF2 | 4.3 | Apoptotic |
| hsa-miR-2861 | -2.96 | BCAM | -2.44 | other function |
| hsa-miR-2861 | -2.96 | CBX5 | -2.2 | other function |
| hsa-miR-23a* | 2.58 | MXRA5 | -2.16 | other function |
| hsa-miR-222* | 1.22 | NAV3 | 2.1 | other function |
| hsa-miR-21* | 1.9 | CDCA7 | -2.5 | other function |
| hsa-miR-21* | 1.9 | GPRC5B | -2.51 | other function |
| hsa-miR-21* | 1.9 | GRHL1 | 3.19 | other function |
| hsa-miR-21* | 1.9 | GRHL1 | 3.19 | other function |
| hsa-miR-21* | 1.9 | NAV2 | -2.08 | other function |
| hsa-miR-21* | 1.9 | NAV2 | -2.08 | other function |
| hsa-miR-1972 | 2.73 | PEG10 | -3.33 | Anti-apoptotic |
| hsa-miR-1972 | 2.73 | KCTD11 | 2.01 | Apoptotic |
| hsa-miR-1972 | 2.73 | TP53 | -2.5 | Apoptotic |
| hsa-miR-1909 | -5.32 | NFIB | -2.07 | other function |
| hsa-miR-146b-5p | 2.73 | MYBL1 | -2.74 | Anti-apoptotic |
| hsa-miR-146b-5p | 2.73 | NR2C2AP | -2.04 | Anti-apoptotic |
| hsa-miR-146b-5p | 2.73 | SOD2 | 2.34 | Anti-apoptotic |
| hsa-miR-146b-5p | 2.73 | PLAUR | 2.76 | Anti-apoptotic |
| hsa-miR-146b-5p | 2.73 | CTNNAL1 | -2.32 | Apoptotic |
| hsa-miR-146b-5p | 2.73 | PLK2 | -2.92 | Apoptotic/Anti-apoptotic |
| hsa-miR-146b-5p | 2.73 | DLK2 | -2.02 | other function |
| hsa-miR-146b-5p | 2.73 | GJB4 | 2.26 | other function |
| hsa-miR-146b-5p | 2.73 | KIF24 | -2.1 | other function |
| hsa-miR-146b-5p | 2.73 | MPHOSPH9 | -2.05 | other function |
| hsa-miR-146b-5p | 2.73 | PER3 | -2.24 | other function |
| hsa-miR-146b-3p | 6.29 | PDZK1IP1 | 3.61 | Anti-apoptotic |
| hsa-miR-146b-3p | 6.29 | IL32 | 2.03 | Apoptotic |
| hsa-miR-146b-3p | 6.29 | PBX1 | -2.48 | Apoptotic |
| hsa-miR-146b-3p | 6.29 | PRDM1 | 8.02 | Apoptotic |
| hsa-miR-146b-3p | 6.29 | SORT1 | 2.14 | Apoptotic |
| hsa-miR-146b-3p | 6.29 | TRIB2 | 2.11 | Apoptotic |
| hsa-miR-146b-3p | 6.29 | ADAM8 | 2.27 | other function |
| hsa-miR-146b-3p | 6.29 | CCDC113 | -2.26 | other function |
| hsa-miR-146b-3p | 6.29 | CITED4 | 2.09 | other function |
| hsa-miR-146b-3p | 6.29 | GLDC | 2.24 | other function |
| hsa-miR-146b-3p | 6.29 | GRHL1 | 3.19 | other function |
| hsa-miR-146b-3p | 6.29 | NAV2 | -2.08 | other function |
| hsa-miR-146b-3p | 6.29 | PLCE1 | -2.15 | other function |
| hsa-miR-146b-3p | 6.29 | SLCO4A1 | 2.12 | other function |
| hsa-miR-146b-3p | 6.29 | ZNF488 | -2.39 | other function |
| hsa-miR-145 | 2.18 | MPP1 | -2.7 | Anti-apoptotic |
| hsa-miR-145 | 2.18 | PEG10 | -3.33 | Anti-apoptotic |
| hsa-miR-145 | 2.18 | PDZK1IP1 | 3.61 | Anti-apoptotic |
| hsa-miR-145 | 2.18 | MUC1 | 2.03 | Anti-apoptotic |
| hsa-miR-145 | 2.18 | FBXO32 | -2.28 | Apoptotic |
| hsa-miR-145 | 2.18 | FLNB | 2.08 | other function |
| hsa-miR-145 | 2.18 | HSD17B2 | 2.4 | other function |
| hsa-miR-145 | 2.18 | JHDM1D | 4.71 | other function |
| hsa-miR-145 | 2.18 | MFSD2A | 2.2 | other function |
| hsa-miR-145 | 2.18 | NAV3 | 2.1 | other function |
| hsa-miR-145 | 2.18 | NFIB | -2.07 | other function |
| hsa-miR-145 | 2.18 | NTN4 | -2.08 | other function |
| hsa-miR-145 | 2.18 | PLCE1 | -2.15 | other function |
| hsa-miR-145 | 2.18 | SQRDL | 2.69 | other function |
| hsa-miR-134 | 1.77 | MYB | -2.23 | Anti-apoptotic |
| hsa-miR-134 | 1.77 | PDZK1IP1 | 3.61 | Anti-apoptotic |
| hsa-miR-134 | 1.77 | SOD2 | 2.34 | Anti-apoptotic |
| hsa-miR-134 | 1.77 | ID1 | 2.65 | Apoptotic/Anti-apoptotic |
| hsa-miR-134 | 1.77 | IL13RA2 | 2.02 | Apoptotic |
| hsa-miR-134 | 1.77 | TAGLN3 | 2.38 | Apoptotic |
| hsa-miR-134 | 1.77 | DHRS9 | 2.73 | other function |
| hsa-miR-134 | 1.77 | MRM1 | -2.02 | other function |
| hsa-miR-134 | 1.77 | RHCG | 2.32 | other function |
| hsa-miR-134 | 1.77 | SDCBP2 | 2.75 | other function |
| hsa-miR-134 | 1.77 | SPRR2A | 6.25 | other function |
| hsa-miR-134 | 1.77 | SQRDL | 2.69 | other function |
| hsa-miR-1231 | -5.14 | MYH10 | -2.04 | other function |
