# Supplementary material for: MicroRNAs Are Part of the Regulatory Network that Controls EGF Induced Apoptosis, Including Elements of the JAK/STAT Pathway, in A431 Cells
Source: PLoS One. 2015 Mar 17;10(3):e0120337. doi: 10.1371/journal.pone.0120337 (PMC4364457; doi:10.1371/journal.pone.0120337)
Supplement: S2 Table — (DOCX) [file pone.0120337.s002.docx]

Supplementary Table 2: Differentially regulated interaction between miRNAs and genes in Proteomics data in A431 cells after EGF treatment.

A- Protein level expression with their target gene(s) miRNA gene expression at 3h.

| miRNA ID | miRNA fold change 3h vs 0h | Target ID | Combined IonScore | Protein fold change3h vs 0h | Apoptotic or anti apoptotic Function |
| --- | --- | --- | --- | --- | --- |
| hsa-miR-762 | -2.04 | WARS | 812 | -1.04 | Other function |
| hsa-miR-675 | -2.27 | HADHA | 675 | -1.04 | Other function |
| hsa-miR-663 | -1.85 | CRABP2 | 249 | -1.1 | Apoptotic |
| hsa-miR-602 | -2.77 | CTSH | 317 | -1.15 | Other function |
| hsa-miR-596 | -2.6 | CFL1 | 984 | -1.27 | Apoptotic |
| hsa-miR-494 | -1.38 | SERPINB3 | 245 | -1.04 | Anti-apoptotic |
| hsa-miR-494 | -1.38 | PDIA3 | 1268 | 1.1 | Apoptotic |
| hsa-miR-432 | -2.04 | TCP1 | 2763 | -1.04 | Other function |
| hsa-miR-3185 | -2.9 | PGK1 | 337 | -1.13 | Other function |
| hsa-miR-29b-1* | 3.75 | ACTB | 357 | -1.06 | Other function |
| hsa-miR-23a* | 1.76 | CTSH | 317 | -1.15 | Other function |
| hsa-miR-1909 | -2.77 | CFL1 | 984 | -1.27 | Apoptotic |
| hsa-miR-149* | -1.93 | CAP1 | 778 | -1.06 | Apoptotic |
| hsa-miR-145 | 2.67 | ACTB | 357 | -1.06 | Other function |
| hsa-miR-134 | 2.39 | CRABP2 | 249 | -1.1 | Apoptotic |
| hsa-miR-134 | 2.39 | BANF1 | 158 | -1.09 | Other function |

B- Protein level expression with their target gene(s) miRNA gene expression at 12h.

| miRNA ID | miRNA fold change 12h vs 0h | Target ID | Combined IonScore | Protein fold change 12h vs 0h | Apoptotic or anti apoptotic Function |
| --- | --- | --- | --- | --- | --- |
| hsa-miR-762 | -3.36 | WARS | 812 | 1.25 | Other function |
| hsa-miR-675 | -2.59 | HADHA | 675 | -1.11 | Other function |
| hsa-miR-663 | -3.19 | CRABP2 | 249 | 1.04 | Apoptotic |
| hsa-miR-602 | -3.49 | CTSH | 317 | -1.53 | Other function |
| hsa-miR-596 | -3.4 | CFL1 | 984 | -1.42 | Apoptotic |
| hsa-miR-494 | -3.68 | SERPINB3 | 245 | 1.14 | Anti-apoptotic |
| hsa-miR-494 | -3.68 | PDIA3 | 1268 | 1.23 | Apoptotic |
| hsa-miR-432 | 2.03 | TCP1 | 2763 | 1.14 | Other function |
| hsa-miR-3185 | -4.44 | PGK1 | 337 | -1.24 | Other function |
| hsa-miR-29b-1* | 2.15 | ACTB | 357 | -1.13 | Other function |
| hsa-miR-23a* | 2.58 | CTSH | 317 | -1.53 | Other function |
| hsa-miR-1909 | -5.32 | CFL1 | 984 | -1.42 | Apoptotic |
| hsa-miR-149* | -2.71 | CAP1 | 778 | 1.22 | Apoptotic |
| hsa-miR-145 | 2.18 | ACTB | 357 | -1.13 | Other function |
| hsa-miR-134 | 1.77 | CRABP2 | 249 | 1.04 | Apoptotic |
| hsa-miR-134 | 1.77 | BANF1 | 158 | -1.15 | Other function |
